# Understanding the role of vanadium: insights into bacterial responses and adaptations

**DOI:** 10.3389/fmicb.2026.1788713

**Published:** 2026-04-09

**Authors:** Joana B. Caldeira, Rita Branco, Paula V. Morais

**Affiliations:** Centre for Mechanical Engineering, Materials and Processes, ARISE, Department of Life Sciences, University of Coimbra, Coimbra, Portugal

**Keywords:** bacterial metal resistance, biomobilization, critical metal, review, vanadate, vanadium, vanadium resistance molecular mechanisms

## Abstract

Vanadium (V) is a critical and important metal used in various industries, but its accumulation in the environment poses a risk and can lead to pollution. The study of microorganisms for metal recycling in renewable biotechnologies has attracted significant research interest. However, there is limited information on the interaction between V and bacteria. The present paper aims to summarize advancements made in the last 5 years (2020–2025) by systematically reviewing articles that mention V. We analyzed a total of 347 articles, ultimately focusing on 45 relevant studies from three different databases. This work enhances our understanding of the bacterial mechanisms responding to V-exposure, as reported in the literature over the last 5 years. The published articles primarily focus on three areas: the exploration of V-containing proteins, the investigation of genes and proteins that are most active with V-exposure, or bioremediation processes. The articles demonstrate a clear that become most active upon V-exposure, and the study of bioremediation processes involving V. The articles illustrate a clear biological relationship between V resistance mechanisms and denitrification processes. Specifically, it has been shown that certain metabolic activities typically associated with nitrates and nitrites become more prevalent in the presence of V. Moreover, mechanisms that provide resistance to other metals, such as chromate and arsenate, are suggested to also contribute to cellular resistance to V. Similar to the effects of other metals, V-exposure appears to induce oxidative stress, with many stress protection mechanisms being enhanced during V-exposure. While some studies indicate that cells can perform V-bioreduction, quantifying this process and making comparisons is challenging due to limitations in experimental design. Extracellular V-immobilization has been observed through interactions with bacterial extracellular polymeric substances; however, the specific enzymatic activities involved remain unidentified. This review also identifies some knowledge gaps that will drive future research into bacterial interactions with V. The lack of identified dedicated V(V)-reductases, as well as unclear mechanisms of V transport and intracellular handling, requires further investigation. By consolidating this information, the review reveals bacterial mechanisms related to V and offers insights for the development of new biotechnologies.

## 1 Introduction

Vanadium (V) has become increasingly relevant in the European Union (EU) industry in recent years and has been classified as “Critical Raw Material” (CRM). CRMs are essential to the EU economy, and a shortage of these materials could disrupt the EU supply chain. They hold considerable importance based on one or more of the following criteria: economic, industrial, or technological (European Commission et al., 2023). In 2023, the EU released an updated list of CRMs, which comprises 34 raw materials, including some metals (European Commission et al., 2023). V is exclusively supplied by four non-EU countries: China (61.6%), Russia (19.8%), South Africa (10.6%), and Brazil (7.6%) (European Commission et al., 2023). The increasing consumption of V by several industries has intensified the demand for this element from these non-EU countries, contributing to its inclusion in the EU’s list of CRMs in 2017 (European Commission et al., 2017).

V is one of the heavy metals highly concentrated in seawater, with concentrations of around 35 nM, mostly as dihydrogen vanadate (Rehder, 2015). In freshwater, the concentration of V is approximately one-third of that found in the oceans (10 nM) (Rehder, 2015). V migration in water and soil occurs mostly with processes of dissolution-precipitation, reduction–oxidation, sorption–desorption and complexation (Huang et al., 2015; Telfeyan et al., 2015). V can also be found in the environment in complex polymetallic ores, such as V titano-magnetite ore (V-Ti mines), which is the major source of V worldwide, accounting for more than 80% (Li and Huang, 2015). Several processes, including various leaching strategies, have been tested and utilized for V-extraction. However, these processes inevitably produce millions of tons of by-product tailings, which are discarded as waste residues (Wang et al., 2019). These residues contain several toxic contaminants that can be released, polluting soils and waters and posing a threat to human health (Yang et al., 2015; Wang et al., 2019; Gan et al., 2023).

V is mostly used as a chemical catalyst and in alloy steels when combined with other metals. The industries that rely on it are diverse and include aerospace/defense, construction, mobility/automotive, and renewable energy (Monakhov et al., 2004; Gustafsson, 2019). V is often released as a residue from industrial activities, which can percolate and contaminate the environment (Monakhov et al., 2004).

This metal may have biological importance and potentially some clinical applications (Rehder, 2015; Gottesman and Mustaev, 2018). Some studies have shown that the behavior of V varies depending on its concentration. Lower concentrations of V can promote biological metabolism, while higher concentrations can be cytotoxic (Zhang et al., 2022; Fei et al., 2023). Heavy metals, such as V, can promote modifications in the microbiological communities and the development of resistance mechanisms within these communities, enabling microorganisms to survive and proliferate despite the presence of toxic metals (Srivastava and Kowshik, 2013).

In recent years, researchers have focused on microorganisms to develop new biotechnologies aimed at recycling heavy metals from contaminated environments. These microorganisms’ abilities to accumulate, leach, or reduce heavy metals has been a key aspect of this research (Coimbra et al., 2017; Caldeira et al., 2021a, 2021b; Farias et al., 2022; Tang et al., 2023). Considering the potential for novel biotechnologies in this research area, studies have been conducted to explore the capacity of microorganisms to remove V(V). This research has involved specific strategies, including bioaccumulation, bioreduction, or the removal of V with different bacterial strains, employing either solutions or residues containing V, as described throughout this review.

The purpose of this review is to systematically survey the existing literature published in the last 5 years (2020–2025) concerning the interactions between V and bacteria, as well as the molecular mechanisms related to V in bacterial systems.

## 2 Methods

To analyze the relevant publications to retrieve, an advanced search combining specific keywords and filters was used on February 8, 2026, to explore the literature present in three different databases: Scopus, Web of Science, and PubMed. The advanced search focused on two primary keywords: “Vanad*” and “bacteri*.” Other keywords were also incorporated as second filter: “gene,” “protein,” “metal resistance,” “reduction” or “bioaccumulation”; to broaden the scope of the analysis to include research involving biological mechanisms of bacteria with exposure to V. This approach explores the interdisciplinary nature of the topic and examines potential bacterial pathways for V. Additionally, another filter was applied to include multiple subject areas, such as agricultural sciences, biochemistry, medicine, health sciences, and multidisciplinary fields. Finally, filters were applied to remove non-English papers and to limit the search to primary research documents as articles.

**Figure 1:**
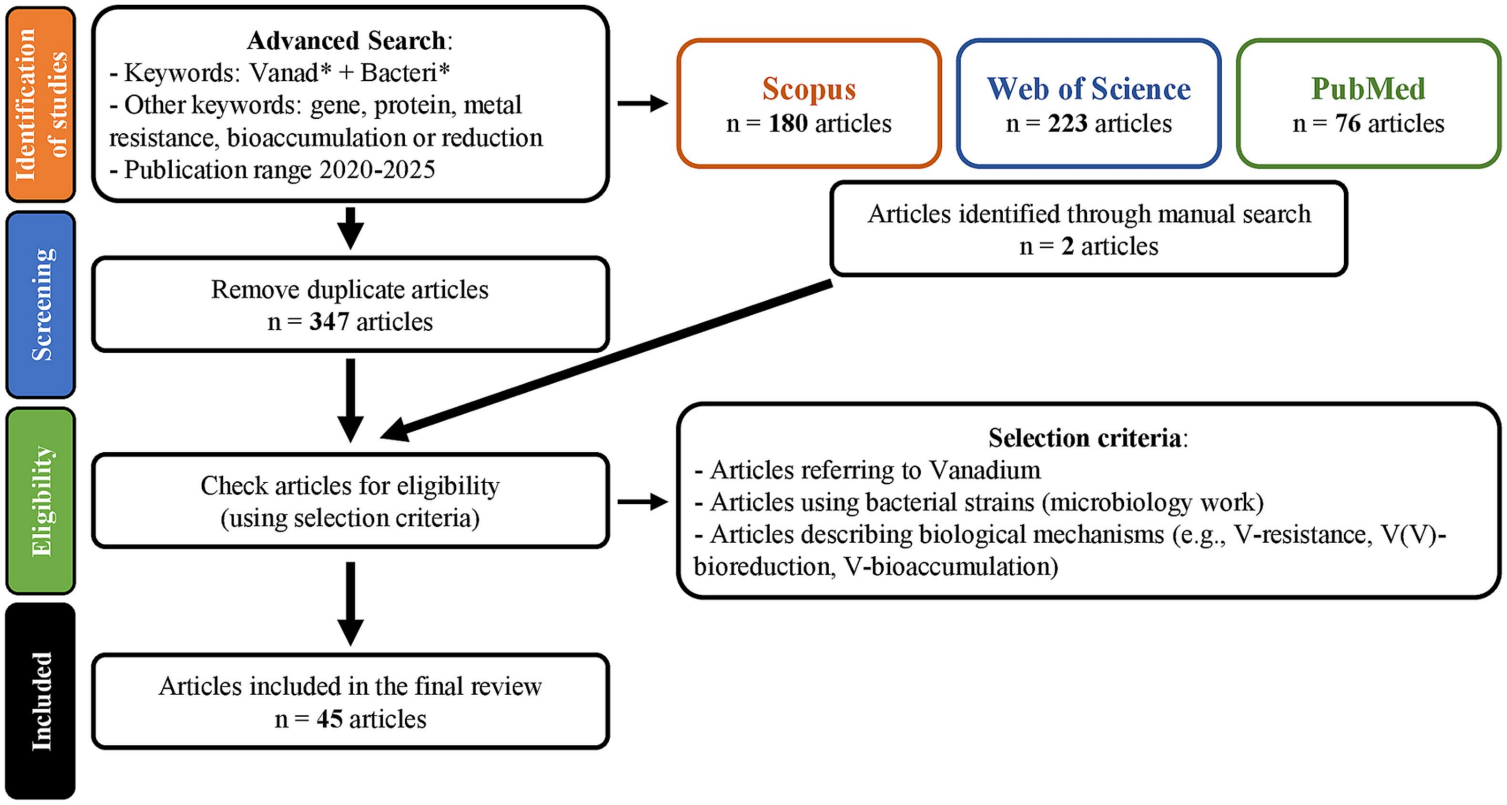
PRISMA flow diagram to illustrate the systematic review process, with the identification of studies, screening and the articles included.

As illustrated in the PRISMA flow diagram (Figure 1), the search strategy identified a total of 180 documents published in Scopus Database, 76 documents in PubMed and 223 documents in Web of Science. The merged list, excluding duplicates, compiled 347 articles. However, it was found that merely 45 of these documents were aligned with the objective of this review (Supplementary Table 1). The selection of documents was focused on articles regarding bacterial strains’ interaction with V (microbiology work), articles referring to genes or proteins (biological mechanisms) involved in this interaction and articles describing biological mechanisms as V-biomobilization (e.g., V-bioreduction, V-bioaccumulation, and V-resistance). However, it is important to note that, although the remaining articles did not fit into this category, 27% of the articles were articles on medical applications, V-nanoparticles or V-complexes; 14% addressed environments containing V or V-contaminated sites (such as V-Ti mine waste or other mines); 7% of the articles describe V-nitrogenases (although not related to V-exposure) and 5% of addressed V as a protein inhibitor (particularly phosphatases).

## 3 Biological use of vanadium

V is a transition metal with two naturally occurring isotopes (51 V and 50 V) and can be found in various valence states, including +3 (V(III)), +4 (V(IV) – Vanadyl), and +5 (V(V) – Vanadate) (Priya et al., 2025). V is in the 4th period of the periodic table, along with other metals, such as arsenic (As), chromium (Cr), nickel (Ni) and selenium (Se). Previous literature described V(V) and arsenate (As(V)) as exhibiting chemical similarities, contaminating similar environments and mimicking phosphate behavior (Ji et al., 2020; Priya et al., 2025). Additionally, V(V) can be considered toxic when it replaces phosphate in certain enzymes and phosphate-dependent biological processes, inhibiting enzymes or halting metabolic processes (Lacabanne et al., 2020; Priya et al., 2025). Various proteins have been reported to be inhibited by V(V), such as, for example, V(V) inhibits Feo-mediated iron transport in *Vibrio cholerae* (Shin et al., 2021), protein oxidase/decarboxylase ScoE in *Streptomyces coeruleorubidus* (Jonnalagadda et al., 2021), a putative ABC-type multidrug efflux transporter RanB in *Riemerella anatipestifer* (Li et al., 2020), alkaline phosphatase (APase) PhoK in *Sphingobium* sp. strain TCM1 (Takahashi et al., 2020), DnaB helicase and a multidrug ATP-transporter BmrA from *Helicobacter pylori* and *Bacillus subtilis*, respectively (Lacabanne et al., 2020).

It has been established that certain organisms can tolerate high concentrations of V, with the ability to withstand concentrations of 5,000 mg/L of V and above (Maltman et al., 2024). This V-tolerance has been identified as a significant characteristic in the description of new species, such as *Shewanella metallivivens*, *Brevundimonas aurifodinae*, and in the functional annotation of the strain *Rhodopseudomonas* AZUL genome (Guardia et al., 2022; Maltman et al., 2023, 2024). Additionally, some organisms may even require V in low concentrations for their metabolism. Enzymes containing V have been identified, such as V-dependent haloperoxidases (VHPO) and V-nitrogenases. VHPOs are found in algae, e.g., *Saccharina japonica* (Yuan et al., 2025), *Ascophyllum nodosum* (Zeides et al., 2025), *Corallina officinalis* (Zeides et al., 2025), *Corallina pilulifera* (Zeides et al., 2025); fungi, e.g., *Hortaea werneckii* UBOCC-A-208029 (Cochereau et al., 2023), *Curvularia inaequalis* (Zeides et al., 2025), and *Penicillium oxalicum* (Priya et al., 2025); archae, e.g., *Methanosarcina acetivorans* (Riyaz and Khan, 2024) and *Methanosarcina barkeri* (Riyaz and Khan, 2024); and a few bacteria, e.g., *Acaryochloris marina* MBIC 11017 (Zeides et al., 2025), *Streptomyces* sp. (Chen et al., 2022), and *Azotobacter chroococcum* (Biełło et al., 2023). VHPOs have a V-ion ligand and can be classified as V-iodoperoxidases (V-IPO), V-chloroperoxidases (V-ClPO), or V-bromoperoxidases (V-BrPO), depending on the oxidized halides (I^−^, Br^−^, Cl^−^) involved (Baumgartner et al., 2024; Zeides et al., 2025). Due to their high stability and tolerance to reaction conditions, VHPO enzymes hold potential for biotechnological applications in industry (Zeides et al., 2025). In the presence of hydrogen peroxide, they primarily catalyze the reaction involving a halide, e.g., in Zeides et al., 2025. In the last 5 years, some studies have explored these enzymes containing V, particularly through structural and biochemical investigations. These investigations have focused on the binding site, halogen oxidation reaction, and the enzymes’ specificity for halogens. However, they do not address the relation between V(V)-exposure and VHPOs presence or activity. While these articles are interesting and provide a strong foundation for further exploration of these enzymes as biocatalysts in the future, they fall outside the scope of this review article.

## 4 Molecular mechanisms of microbial interaction with vanadium

Previous studies aimed to explore the bacterial mechanisms that promote resistance to V-exposure, with a particular focus on V(V)-bioreduction (Table 1). However, the limited existing literature provides detailed information on specific genes and proteins associated with V (Zeides et al., 2025). Nevertheless, studies have characterized “V Detoxifying Genes” (VDG) as functional genes that encode: (1) proteins that modify the valence state of the metal (nitrate and sulfate reduction genes), (2) proteins that promote the efflux of heavy metals from cells (heavy metal efflux pumps and ABC transporters), and (3) proteins that are involved in the repair of oxidative damage induced by heavy metals (antioxidant enzyme systems) (Xia et al., 2022). Of the articles extracted using advanced database searches, approximately 40 articles described V-contamination environments and searched for VDG in the metagenome, but did not explore the V-effect on gene expression or abundance. In the absence of a demonstrable relationship between gene expression or abundance and V-exposure, the referred articles were not included in the review with a view to examining bacterial mechanisms.

**Table 1:** List of the possible bacterial V detoxifying mechanisms from information provided in reported studies: molecular mechanisms and the genes involved, bacterial strains and the sample origin, experimental evidence and analysis, and condition tested (techniques reported in the cited studies).

Molecular mechanism	Gene	Bacterial strain(s)	Experimental evidence	Analysis	Sample/conditions	Sample origin	Reference
Bacillithiol synthesis	*bshB2*	*Bacillus megaterium* R01	RT-qPCR (RNA analysis)	Comparing the gene abundances obtained with and without V(V)	Exposure to different V(V) concentrations for 1 day (24 h) at 30 °C	Strain isolated from a stone coal mine region in Yiyang City, Hunan Province (China)	Huang et al. (2025)
	*bshC*	*Bacillus megaterium* R01	RT-qPCR (RNA analysis)	Comparing the gene abundances obtained with and without V(V)	Exposure to different V(V) concentrations for 1 day (24 h) at 30 °C	Strain isolated from a stone coal mine region in Yiyang City, Hunan Province (China)	Huang et al. (2025)
Bacillithiol reductase	*ypdA*	*Bacillus megaterium* R01	RT-qPCR (RNA analysis)	Comparing the gene abundances obtained with and without V(V)	Exposure to different V(V) concentrations for 1 day (24 h) at 30 °C	Strain isolated from a stone coal mine region in Yiyang City, Hunan Province (China)	Huang et al. (2025)
Bacilliredoxin monothiol	*ytxJ*	*Bacillus megaterium* R01	RT-qPCR (RNA analysis)	Comparing the gene abundances obtained with and without V(V)	Exposure to different V(V) concentrations for 1 day (24 h) at 30 °C	Strain isolated from a stone coal mine region in Yiyang City, Hunan Province (China)	Huang et al. (2025)
Carbonic anhydrase (CA)		*Bacillus mucilaginosus*	Spectrophotometric method	Comparing enzyme activity per protein with and without V(V)	Exposure to 10 mg/L V(V) for 5 days (120 h) at 30 °C	Bacterial strain mutagenized	Dong et al. (2024)
Catalase		*Enterococcus faecalis*	Quantification enzyme activity	Comparing enzyme units per cell with and without V(V)	Incubation with 250 mg/L V(V) in anaerobic conditions for 7 days at 30 °C	Strain isolated from V-contaminated soils	Zhang L. et al. (2025)
		*Pseudogulbenkiania* sp. 2002	Quantification enzyme activity	Comparing enzyme units per cell with and without V(V)	Incubation with 10 mg/L V(V) in anaerobic conditions for 5 days (120 h) at 30 °C	Strain isolated from freshwater lake sediments	Fei et al. (2023)*
		*Acidovorax* sp. BoFeN1	Quantification enzyme activity	Comparing enzyme units per cell with and without V(V)	Incubation with 10 mg/L V(V) in anaerobic conditions for 5 days (120 h) at 30 °C	Strain isolated from lake coastal sediments	Fei et al. (2023)*
		*Lactococcus raffinolactis*	Quantification enzyme activity	Comparing enzyme units per protein with and without V(V)	Exposure to V(V) for 10 days at 30 °C	Strain provided by Inner Mongolia Agricultural University (China)	Zhang B. et al. (2021)*
Cation diffusion facilitator/cobalt–zinc–cadmium transporter (metal resistance)	*czcD*	*Microbacterium liquefaciens* MNSH1-9 K-1	RT-qPCR (RNA analysis)	Comparing the gene expression under exposure to V and Ni: 2-fold change in expression with 12 h and an increase in gene expression with 24 h	Exposure to 200 ppm V and Ni at 30 °C	Strain isolated from environmental samples (tailings, soils, sediments, and water) from Guanajuato (Mexico)	Fierros-Romero et al. (2020)
		*Anaeromyxobacter* sp. bin.29	Metagenomic analysis (Illumina Hiseq)	Comparing the distribution of genes in functional genera between the inoculum and the B-S	Columns with continuous flow for 226 days	Aquifer sediments from a V-tailing reservoir (China)	Yan et al. (2023)
		*UBA6901* sp. bin.68					
Chromate transport protein	*chrA*	*Achromobacter* sp. bin.24	Metagenomic analysis (Illumina Hiseq)	Comparing the distribution of genes in functional genera between the inoculum and the B-S	Columns with continuous flow for 226 days	Aquifer sediments from a V-tailing reservoir (China)	Yan et al. (2023)
		*Thiomonas* sp. bin.22					
		*Thiomonas* sp. bin.61					
		*Thiomonas arsenitoxydans* bin.5					
		Microbial community (*Deltaproteobacteria, Anaerolineae, Campylobacteria, Bacteroidia* and *Gammaproteobacteria*)	RT-qPCR (RNA analysis)	Comparing the gene abundances obtained	Columns with anaerobic microbial community for 276 days at 22 °C	Brewery wastewater treatment (China)	Shi et al. (2020)
	*chrAC*	*Sulfuricurvum* sp. bin.38	Metagenomic analysis (Illumina Hiseq)	Comparing the distribution of genes in functional genera between the inoculum and the B-S	Columns with continuous flow for 226 days	Aquifer sediments from a V-tailing reservoir (China)	Yan et al. (2023)
		*Pseudomonas cremoris* bin.59					
		*Anaeromyxobacter* sp. bin.29					
	*chrC*	*Methanobacterium* sp. bin.62	Metagenomic analysis (Illumina Hiseq)	Comparing the distribution of genes in functional genera between the inoculum and the B-S	Columns with continuous flow for 226 days	Aquifer sediments from a V-tailing reservoir (China)	Yan et al. (2023)
		*UBA6901* sp. bin.68					
Cytochrome c		Chemoautotrophic genera (e.g., *Thiobacillus*)	Quantification enzyme activity	Comparing enzyme activity per protein with and without V(V)	Columns with continuous flow for 150 days at 22 °C	Anaerobic consortium from brewery wastewater treatment	He et al. (2021)
		*Acidovorax* sp. BoFeN1	Quantification enzyme assay	Comparing enzyme activity per protein with and without V(V)	Exposure to 10 mg/L V(V) for 5 days (120 h) at 30 °C	Strain isolated from Lake Constance coastal sediments	Fei et al. (2024) and Zhang B. et al. (2025)*
		*Pseudogulbenkiania* sp. 2002	Quantification enzyme assay	Comparing concentration with and without V(V)	Incubation with V(V)	Strain isolated from freshwater lake sediments	Zhang B. et al. (2025)*
		Microbial community (e.g., *Thiobacillus, Desulfovibrio* and *Thermomonas*)	Quantification enzyme activity	Comparing enzyme activity per protein with Control	Anaerobic system with solution periodically refreshed in operational cycles of 7 days	Aquifer sediments from V production region (China)	He et al. (2023)
		Microbial community (e.g., *Syntrophobacter*, *Spirochaeta*, *Geobacter*)	Quantification enzyme activity	Comparing enzyme activity per protein with and without V(V)	Bioreactors with three consecutive operating cycles and V(V) exposure	Anaerobic sludge collected from a brewery anaerobic system without V(V) exposure (China)	Wang et al. (2021)
Cytochrome c maturation protein C	*ccmC*	*Methanobacterium* sp. bin.62	Metagenomic analysis (Illumina Hiseq)	Comparing the distribution of genes in functional genera between the inoculum and the B-S	Columns with continuous flow for 226 days	Aquifer sediments from a V-tailing reservoir (China)	Yan et al. (2023)
Cytochrome c nitrite reductase protein	*nrfA*	*Shewanella* sp. trx1	Quantification of enzyme activity and RT-qPCR (RNA analysis)	Comparing the gene abundances obtained and the enzyme activity with and without V(V)	Exposure to 610 mg/L V(V) for 1 day (24 h) at 30 °C		Cheng et al. (2025)
Cytoplasmic membrane component	*cymA*	*Anaeromyxobacter* sp. bin.29	Metagenomic analysis (Illumina Hiseq)	Comparing the distribution of genes in functional genera between the inoculum and the B-S	Columns with continuous flow for 226 days	Aquifer sediments from a V-tailing reservoir (China)	Yan et al. (2023)
		*UBA6901* sp. bin.68					
		*Polaromonas* spp.	Metagenomic analysis (Illumina Hiseq)	Focusing especially on the putative V(V) reducing bacteria	set of anaerobic enrichment cultures, incubation for 49 days	Tailings from an active V mine in Chenxi County, Hunan (China)	Sun et al. (2020)
Cysteine synthesis	*cysJ*	*Bacillus megaterium* R01	RT-qPCR (RNA analysis)	Comparing the gene abundances obtained with and without V(V)	Exposure to different V(V) concentrations for 1 day (24 h) at 30 °C	Strain isolated from a stone coal mine region in Yiyang City, Hunan Province (China)	Huang et al. (2025)
Extracellular polymeric substances (EPS)		*Bacillus subtilis*	FTIR spectroscopy (functional group characterization)		Growth with 50 mg/L V(V) for 10 days at 30 °C	Strain provided by Fujian Agriculture and Forestry University (China)	Yan et al. (2025)*
		*Thauera humireducens*	FTIR spectroscopy (functional group characterization)		Growth with 50 mg/L V(V) for 10 days at 30 °C	Strain provided by Fujian Agriculture and Forestry University (China)	Yan et al. (2025)*
		*Acidovorax* sp. BoFeN1	FTIR spectroscopy (functional group characterization)		Exposure to 10 mg/L V(V) for 5 days (120 h) at 30 °C	Strain isolated from Lake Constance coastal sediments	Fei et al. (2024)*
		Microbial community (*Acinetobacter, Dechlorobacter, Denitratisoma* and *Nitrospira*)	Metagenomic analysis (Illumina Novaseq) and TEM	Focusing especially on the putative V(V) reducing bacteria	Biofilm reactor with continuous flow for 100 days at 24 °C	Membrane-aerated biofilm reactor system	Sun et al. (2025)
		Microbial community (e.g., *Syntrophobacter*, *Spirochaeta*, *Geobacter*)	Quantification method	Comparing enzyme activity per protein with and without V(V)	Bioreactors with three consecutive operating cycles and V(V) exposure	Anaerobic sludge collected from a brewery anaerobic system without V(V) exposure (China)	Wang et al. (2021)
		*Bacillus* sp. PFYN01	FTIR spectroscopy (functional group characterization) and 3DEEM fluorescence spectroscopy (determination of interaction EPS-V)	Analyzing EPS groups characterization and comparing the distribution of V(V)	Incubation with 0 to 100 mg/L V(V) for 0.5 days (12 h) at 30 °C	Strain isolated from Majiatian V tailing reservoir in Panzhihua (China)	Zhou et al. (2022)*
		Thermophilic hydrogen-producing bacteria (*Bacilli* and *Clostridia*)	3DEEM fluorescence spectroscopy (determination of interaction EPS-V)	Analyzing EPS and comparing the distribution of V(V)	Anaerobic sludge enriched for 60 days at 55 °C	Sample collected from anaerobic wastewater treatment station in Rizhao, Shandong (China)	Zheng et al. (2021)
		*Enterococcus faecalis*	3DEEM fluorescence spectroscopy (determination of interaction EPS-V) and TEM	Analyzing EPS and comparing the distribution of V(V)	Incubation with 250 mg/L V(V) in anaerobic conditions for 7 days at 30 °C	Strain isolated from V-contaminated soils	Zhang L. et al. (2025)*
Membrane cytochrome	*mtrA*	*Sulfuricurvum* sp. bin.38	Metagenomic analysis (Illumina Hiseq)	Comparing the distribution of genes in functional genera between the inoculum and the B-S	Columns with continuous flow for 226 days	Aquifer sediments from a V-tailing reservoir (China)	Yan et al. (2023)
		*Pseudomonas cremoris* bin.59					
		*Anaeromyxobacter* sp. bin.29					
		*UBA6901* sp. bin.68					
		*Achromobacter* sp. bin.24					
		*Thiomonas* sp. bin.22					
		*Thiomonas* sp. bin.61					
		*Thiomonas arsenitoxydans* bin.5					
	*mtrC*	Microbial community (*Deltaproteobacteria, Anaerolineae, Campylobacteria, Bacteroidia* and *Gammaproteobacteria*)	RT-qPCR (RNA analysis)	Comparing the gene abundances obtained	Columns with anaerobic microbial community for 276 days at 22 °C	Brewery wastewater treatment (China)	Shi et al. (2020)
Metal-binding metallothionein	*smtB*	*Anaeromyxobacter* sp. bin.29	Metagenomic analysis (Illumina Hiseq)	Comparing the distribution of genes in functional genera between the inoculum and the B-S	Columns with continuous flow for 226 days	Aquifer sediments from a V-tailing reservoir (China)	Yan et al. (2023)
Methionine ABC transporter		*Ochrobactrum tritici* 5bvl1	SDS-PAGE (analysis of protein profiles) and mass spectrometry (for protein identification)	Comparing protein profiles with and without V(V) and identifying the protein	Growth with 15 mM V(V) for 1 day (24 h) at 30 °C	Strain isolated from activated sludge in a Cr-contaminated area (Portugal)	Almeida et al. (2020)
Nicotinamide adenine dinucleotide (NADH)-dependent reductas		Thermophilic hydrogen-producing bacteria (*Bacilli* and *Clostridia*)	3DEEM fluorescence spectroscopy	Comparing the relative quenching of fluorescence	Anaerobic sludge enriched for 60 days at 55 °C	Sample collected from anaerobic wastewater treatment station in Rizhao, Shandong (China)	Zheng et al. (2021)
		Microbial community (e.g., *Syntrophobacter*, *Spirochaeta*, *Geobacter*)	Quantification enzyme activity	Comparing enzyme activity per protein with and without V(V)	Bioreactors with three consecutive operating cycles and V(V) exposure	Anaerobic sludge collected from a brewery anaerobic system without V(V) exposure (China)	Wang et al. (2021)
		*Bacillus subtilis*	Spectrophotometric method (intracellular NADH quantification)	Comparing abundances with and without V(V)	Growth with 50 mg/L V(V) for 10 days at 30 °C	Strain provided by Fujian Agriculture and Forestry University (China)	Yan et al. (2025)*
		*Acidovorax* sp. BoFeN1	3DEEM fluorescence spectroscopy	Comparing the relative quenching of fluorescence	Exposure to 10 mg/L V(V) for 5 days (120 h) at 30 °C	Strain isolated from Lake Constance coastal sediments	Fei et al. (2024)*
		*Enterococcus faecalis*	Quantification enzyme	Comparing enzyme units per cell with and without V(V)	Incubation with 250 mg/L V(V) in anaerobic conditions for 7 days at 30 °C	Strain isolated from V-contaminated soils	Zhang L. et al. (2025)*
		*Lactococcus raffinolactis*	Spectrophotometric method (intracellular NADH quantification)	Comparing enzyme activity with and without V(V)	Exposure to V(V) for 10 days at 30 °C	Strain provided by Inner Mongolia Agricultural University (China)	Zhang B. et al. (2021)*
		Microbial community (e.g., *Thiobacillus, Desulfovibrio* and *Thermomonas*)	Quantification enzyme activity	Comparing enzyme activity per protein with Control	Anaerobic system with solution periodically refreshed in operational cycles of 7 days	Aquifer sediments from V production region (China)	He et al. (2023)
		Chemoautotrophic genera (e.g., *Thiobacillus*)	Spectrophotometric method (intracellular NADH quantification)	Comparing enzyme activity with and without V(V)	Columns with continuous flow for 150 days at 22 °C	Anaerobic consortium from brewery wastewater treatment	He et al. (2021)
Nitrate reductase	*napA*	*Acidovorax* sp. BoFeN1	RT-qPCR (RNA analysis)	Comparing the number of RNA copies per RNA with and without V(V)	Exposure to 10 mg/L V(V) for 5 days (120 h) at 30 °C	Strain isolated from Lake Constance coastal sediments	Fei et al. (2024)
		*Pseudogulbenkiania* sp. 2002	RT-qPCR (RNA analysis)	Comparing the number of RNA copies per RNA with and without V(V)	Incubation with 10 mg/L V(V) in anaerobic conditions for 5 days (120 h) at 30 °C	Strain isolated from freshwater lake sediments	Fei et al. (2023)
		Microbial community (e.g., *Syntrophobacter*, *Spirochaeta*, *Geobacter*)	RT-qPCR (RNA analysis)	Comparing the number of RNA copies per RNA with and without V(V)	Bioreactors with three consecutive operating cycles and V(V) exposure	Anaerobic sludge collected from a brewery anaerobic system without V(V) exposure (China)	Wang et al. (2021)
		Chemoautotrophic genera (e.g., *Thiobacillus*)	Metagenomic analysis (Illumina Miseq) and qPCR (DNA analysis)	Comparing the number of gene copies per DNA with and without V(V)	Columns with continuous flow for 150 days at 22 °C	Anaerobic consortium from brewery wastewater treatment	He et al. (2021)
		*Lactococcus raffinolactis*	qPCR (DNA analysis)	Comparing the number of gene copies per DNA	Exposure to 25, 50, 75 and 100 mg/L V(V) for 10 days at 30 °C	Strain provided by Inner Mongolia Agricultural University (China)	Zhang B. et al. (2021)
		*Sulfuricurvum* sp. bin.38	Metagenomic analysis (Illumina Hiseq)	Comparing the distribution of genes in functional genera between the inoculum and the B-S	Columns with continuous flow for 226 days	Aquifer sediments from a V-tailing reservoir (China)	Yan et al. (2023)
		*UBA6901* sp. bin.68					
		Microbial community (e.g., *Thiobacillus, Desulfovibrio* and *Thermomonas*)	RT-qPCR (RNA analysis)	Comparing the number of RNA copies per RNA with Control	Anaerobic system with solution periodically refreshed in operational cycles of 7 days	Aquifer sediments from V production region (China)	He et al. (2023)
	*napAB*	Microbial community (*Acinetobacter, Dechlorobacter, Denitratisoma* and *Nitrospira*)	Metagenomic analysis (Illumina Novaseq)	Focusing especially on the putative V(V) reducing bacteria	Biofilm reactor with continuous flow for 100 days at 24 °C	Membrane-aerated biofilm reactor system	Sun et al. (2025)
	*napABC*	Microbial community (*Nocardioides, Lysobacter, Sphingomonas* and *Marmoricola*)	Metagenomic analysis (Illumina Miseq)	Comparing the relative abundance of genes after incubation with different V(V) concentrations	Soils exposed to different V(V) concentrations (0, 0.2, 0.5, 0.8, and 1.1 g V/kg soil)	Soils from the Jiangan campus of Sichuan University in Chengdu (China)	Liu et al. (2023)
	*narG*	*Lactococcus raffinolactis*	qPCR (DNA analysis)	Comparing the number of gene copies per DNA	Exposure to 25, 50, 75 and 100 mg/L V(V) for 10 days at 30 °C	Strain provided by Inner Mongolia Agricultural University (China)	Zhang B. et al. (2021)
		*Acidovorax* sp. BoFeN1	RT-qPCR (RNA analysis)	Comparing the number of RNA copies per RNA with and without V(V)	Exposure to 10 mg/L V(V) for 5 days (120 h) at 30 °C	Strain isolated from Lake Constance coastal sediments	Fei et al. (2024)
		*Achromobacter* sp. bin.24	Metagenomic analysis (Illumina Hiseq)	Comparing the distribution of genes in functional genera between the inoculum and the B-S	Columns with continuous flow for 226 days	Aquifer sediments from a V-tailing reservoir (China)	Yan et al. (2023)
		*Thiomonas* sp. bin.22					
		*Thiomonas* sp. bin.61					
		*Thiomonas arsenitoxydans* bin.5					
		*Polaromonas* spp.	Metagenomic analysis (Illumina Hiseq)	Focusing especially on the putative V(V) reducing bacteria	set of anaerobic enrichment cultures, incubation for 49 days	Tailings from an active V mine in Chenxi County, Hunan (China)	Sun et al. (2020)
(NAR)		Chemoautotrophic genera (e.g., *Thiobacillus*)	Quantification enzyme activity	Comparing enzyme activity per protein with and without V(V)	Columns with continuous flow for 150 days at 22 °C	Anaerobic consortium from brewery wastewater treatment	He et al. (2021)
		*Pseudogulbenkiania* sp. 2002	Quantification enzyme activity	Comparing enzyme units per cell with and without V(V)	Incubation with 10 mg/L V(V) in anaerobic conditions for 5 days (120 h) at 30 °C	Strain isolated from freshwater lake sediments	Fei et al. (2023)*
		*Acidovorax* sp. BoFeN1	Quantification enzyme activity	Comparing enzyme units per cell with and without V(V)	Incubation with 10 mg/L V(V) in anaerobic conditions for 5 days (120 h) at 30 °C	Strain isolated from lake coastal sediments	Fei et al. (2023)*
		Microbial community (e.g., *Thiobacillus, Desulfovibrio* and *Thermomonas*)	Quantification enzyme activity	Comparing enzyme activity per protein with Control	Anaerobic system with solution periodically refreshed in operational cycles of 7 days	Aquifer sediments from V production region (China)	He et al. (2023)
(NAP)		*Acidovorax* sp. BoFeN1	Quantification enzyme activity kit	Comparing enzyme activity with and without V(V)	Exposure to 10 mg/L V(V) for 5 days (120 h) at 30 °C	Strain isolated from Lake Constance coastal sediments	Fei et al. (2023, 2024)*
		*Pseudogulbenkiania* sp. 2002	Quantification enzyme activity	Comparing enzyme units per cell with and without V(V)	Incubation with 10 mg/L V(V) in anaerobic conditions for 5 days (120 h) at 30 °C	Strain isolated from freshwater lake sediments	Fei et al. (2023)*
Nitrite reductase	*nirB*	*Pseudomonas balearica* 1A00204	Transcriptomic analysis (differential gene expression)	Comparing the gene expression with and without V(V)	Growth with 1 mM V(V) for 0.5 days (12 h) at 28 °C	Strain provided by the Marine Culture Collection of China (MCCC)	Gan et al. (2024)
	*nirD*	*Pseudomonas balearica* 1A00204	Transcriptomic analysis (differential gene expression)	Comparing the gene expression with and without V(V)	Growth with 1 mM V(V) for 0.5 days (12 h) at 28 °C	Strain provided by the Marine Culture Collection of China (MCCC)	Gan et al. (2024)
	*nirK*	*Acidovorax* sp. BoFeN1	RT-qPCR (RNA analysis)	Comparing the number of RNA copies per RNA with and without V(V)	Exposure to 10 mg/L V(V) for 5 days (120 h) at 30 °C	Strain isolated from Lake Constance coastal sediments	Fei et al. (2023, 2024)
		*Pseudogulbenkiania* sp. 2002	RT-qPCR (RNA analysis)	Comparing the number of RNA copies per RNA with and without V(V)	Incubation with 10 mg/L V(V) in anaerobic conditions for 5 days (120 h) at 30 °C	Strain isolated from freshwater lake sediments	Fei et al. (2023)
	*nirKS*	Microbial community (*Acinetobacter, Dechlorobacter, Denitratisoma* and *Nitrospira*)	Metagenomic analysis (Illumina Novaseq)	Focusing especially on the putative V(V) reducing bacteria	Biofilm reactor with continuous flow for 100 days at 24 °C	Membrane-aerated biofilm reactor system	Sun et al. (2025)
	*nirS*	*UBA6901* sp. bin.68	Metagenomic analysis (Illumina Hiseq)	Comparing the distribution of genes in functional genera between the inoculum and the B-S	Columns with continuous flow for 226 days	Aquifer sediments from a V-tailing reservoir (China)	Yan et al. (2023)
		Microbial community (e.g., *Syntrophobacter*, *Spirochaeta*, *Geobacter*)	RT-qPCR (RNA analysis)	Comparing the number of RNA copies per RNA with and without V(V)	Bioreactors with three consecutive operating cycles and V(V) exposure	Anaerobic sludge collected from a brewery anaerobic system without V(V) exposure (China)	Wang et al. (2021)
		*Pseudogulbenkiania* sp. 2002	RT-qPCR (RNA analysis)	Comparing the number of RNA copies per RNA with and without V(V)	Incubation with 10 mg/L V(V) in anaerobic conditions for 5 days (120 h) at 30 °C	Strain isolated from freshwater lake sediments	Fei et al. (2023)
		*Acidovorax* sp. BoFeN1	RT-qPCR (RNA analysis)	Comparing the number of RNA copies per RNA with and without V(V)	Incubation with 10 mg/L V(V) in anaerobic conditions for 5 days (120 h) at 30 °C	Strain isolated from lake coastal sediments	Fei et al. (2023)
		*Lactococcus raffinolactis*	qPCR (DNA analysis)	Comparing the number of gene copies per DNA	Exposure to 25, 50, 75 and 100 mg/L V(V) for 10 days at 30 °C	Strain provided by Inner Mongolia Agricultural University (China)	Zhang B. et al. (2021)
		Microbial community (e.g., *Thiobacillus, Desulfovibrio* and *Thermomonas*)	RT-qPCR (RNA analysis)	Comparing the number of RNA copies per RNA with Control	Anaerobic system with solution periodically refreshed in operational cycles of 7 days	Aquifer sediments from V production region (China)	He et al. (2023)
(NIR)		*Lactococcus raffinolactis*	Quantification enzyme activity	Comparing enzyme units per protein with and without V(V)	Exposure to V(V) for 10 days at 30 °C	Strain provided by Inner Mongolia Agricultural University (China)	Zhang B. et al. (2021)*
		*Pseudogulbenkiania* sp. 2002	Quantification enzyme activity	Comparing enzyme units per cell with and without V(V)	Incubation with 10 mg/L V(V) in anaerobic conditions for 5 days (120 h) at 30 °C	Strain isolated from freshwater lake sediments	Fei et al. (2023)*
		*Acidovorax* sp. BoFeN1	Quantification enzyme activity kit	Comparing enzyme activity with and without V(V)	Exposure to 10 mg/L V(V) for 5 days (120 h) at 30 °C	Strain isolated from Lake Constance coastal sediments	Fei et al. (2023, 2024)*
		Chemoautotrophic genera (e.g., *Thiobacillus*)	Quantification enzyme activity	Comparing enzyme activity per protein with and without V(V)	Columns with continuous flow for 150 days at 22 °C	Anaerobic consortium from brewery wastewater treatment	He et al. (2021)
		Microbial community (e.g., *Thiobacillus, Desulfovibrio* and *Thermomonas*)	Quantification enzyme activity	Comparing enzyme activity per protein with Control	Anaerobic system with solution periodically refreshed in operational cycles of 7 days	Aquifer sediments from V production region (China)	He et al. (2023)
		*Bacillus* sp. PFYN01	Quantification enzyme NIR kit	Comparing enzyme units per intact cell with and without V(V)	Incubation with 0 to 100 mg/L V(V) for 0.5 days (12 h) at 30 °C	Strain isolated from Majiatian V tailing reservoir in Panzhihua (China)	Zhou et al. (2022)*
Nitric oxidoreductase (NOR)		*Acidovorax* sp. BoFeN1	Quantification enzyme activity kit	Comparing enzyme activity with and without V(V)	Exposure to 10 mg/L V(V) for 5 days (120 h) at 30 °C	Strain isolated from Lake Constance coastal sediments	Fei et al. (2024)*
		*Pseudogulbenkiania* sp. 2002	Quantification enzyme activity	Comparing enzyme units per cell with and without V(V)	Incubation with 10 mg/L V(V) in anaerobic conditions for 5 days (120 h) at 30 °C	Strain isolated from freshwater lake sediments	Fei et al. (2023)*
	*norBC*	Microbial community (*Acinetobacter, Dechlorobacter, Denitratisoma* and *Nitrospira*)	Metagenomic analysis (Illumina Novaseq)	Focusing especially on the putative V(V) reducing bacteria	Biofilm reactor with continuous flow for 100 days at 24 °C	Membrane-aerated biofilm reactor system	Sun et al. (2025)
Nitrous oxidoreductase	*nosZ*	Microbial community (*Acinetobacter, Dechlorobacter, Denitratisoma* and *Nitrospira*)	Metagenomic analysis (Illumina Novaseq)	Focusing especially on the putative V(V) reducing bacteria	Biofilm reactor with continuous flow for 100 days at 24 °C	Membrane-aerated biofilm reactor system	Sun et al. (2025)
		Microbial community (*Nocardioides, Lysobacter, Sphingomonas* and *Marmoricola*)	Metagenomic analysis (Illumina Miseq)	Comparing the relative abundance of genes after incubation with different V(V) concentrations	Soils exposed to different V(V) concentrations (0, 0.2, 0.5, 0.8, and 1.1 g V/kg soil)	Soils from the Jiangan campus of Sichuan University in Chengdu (China)	Liu et al. (2023)
		*Pseudogulbenkiania* sp. strain 2002	RT-qPCR (RNA analysis)	Comparing the number of RNA copies per RNA with and without V(V)	Incubation with 10 mg/L V(V) in anaerobic conditions for 5 days (120 h) at 30 °C	Strain isolated from freshwater lake sediments	Fei et al. (2023)
		*Acidovorax* sp. BoFeN1	RT-qPCR (RNA analysis)	Comparing the number of RNA copies per RNA with and without V(V)	Exposure to 10 mg/L V(V) for 5 days (120 h) at 30 °C	Strain isolated from Lake Constance coastal sediments	Fei et al. (2023, 2024)
	*nxrAB*	Microbial community (*Nocardioides, Lysobacter, Sphingomonas* and *Marmoricola*)	Metagenomic analysis (Illumina Miseq)	Comparing the relative abundance of genes after incubation with different V(V) concentrations	Soils exposed to different V(V) concentrations (0, 0.2, 0.5, 0.8, and 1.1 g V/kg soil)	Soils from the Jiangan campus of Sichuan University in Chengdu (China)	Liu et al. (2023)
(NOS)		*Acidovorax* sp. BoFeN1	Quantification enzyme activity kit	Comparing enzyme activity with and without V(V)	Exposure to 10 mg/L V(V) for 5 days (120 h) at 30 °C	Strain isolated from Lake Constance coastal sediments	Fei et al. (2023, 2024)*
		*Pseudogulbenkiania* sp. 2002	Quantification enzyme activity	Comparing enzyme units per cell with and without V(V)	Incubation with 10 mg/L V(V) in anaerobic conditions for 5 days (120 h) at 30 °C	Strain isolated from freshwater lake sediments	Fei et al. (2023)*
Outer membrane cytochrome	*omcA*	*Anaeromyxobacter* sp. bin.29	Metagenomic analysis (Illumina Hiseq)	Comparing the distribution of genes in functional genera between the inoculum and the B-S	Columns with continuous flow for 226 days	Aquifer sediments from a V-tailing reservoir (China)	Yan et al. (2023)
		*Polaromonas* spp.	Metagenomic analysis (Illumina Hiseq)	Focusing especially on the putative V(V) reducing bacteria	set of anaerobic enrichment cultures, incubation for 49 days	Tailings from an active V mine in Chenxi County, Hunan (China)	Sun et al. (2020)
	*omcAB*	Microbial community (*Deltaproteobacteria, Anaerolineae, Campylobacteria, Bacteroidia* and *Gammaproteobacteria*)	RT-qPCR (RNA analysis)	Comparing the gene abundances obtained	Columns with anaerobic microbial community for 276 days at 22 °C	Brewery wastewater treatment (China)	Shi et al. (2020)
	*omcB*	*Sulfuricurvum* sp. bin.38	Metagenomic analysis (Illumina Hiseq)	Comparing the distribution of genes in functional genera between the inoculum and the B-S	Columns with continuous flow for 226 days	Aquifer sediments from a V-tailing reservoir (China)	Yan et al. (2023)
		*Pseudomonas cremoris* bin.59					
		*Anaeromyxobacter* sp. bin.29					
		*UBA6901* sp. bin.68					
		*Achromobacter* sp. bin.24					
		*Thiomonas* sp. bin.22					
		*Thiomonas* sp. bin.61					
		*Thiomonas arsenitoxydans* bin.5					
		Chemoautotrophic genera (e.g., *Thiobacillus*)	Metagenomic analysis (Illumina Miseq) and qPCR (DNA analysis)	Comparing the number of gene copies per DNA with and without V(V)	Columns with continuous flow for 150 days at 22 °C	Anaerobic consortium from brewery wastewater treatment	He et al. (2021)
Polymyxin-resistance genes	*pmrE*	*Escherichia coli*	Minimum inhibitory concentration	Comparing resistance between different mutated strains	mutated *Escherichia coli* strains	Gene identified from sediment and seawater microbiome	Joo et al. (2023)*
	*pmrF*	*Escherichia coli*	Minimum inhibitory concentration	Comparing resistance between different mutated strains	mutated *Escherichia coli* strains	Gene identified from sediment and seawater microbiome	Joo et al. (2023)*
Quinones		*Acidovorax* sp. BoFeN1	HPLC analysis (metabolite quantification) and quantification enzyme assay	Comparing concentration with and without V(V)	Exposure to 10 mg/L V(V) for 5 days (120 h) at 30 °C	Strain isolated from Lake Constance coastal sediments	Fei et al. (2024) and Zhang B. et al. (2025)
		*Pseudogulbenkiania* sp. 2002	Quantification enzyme assay	Comparing concentration with and without V(V)	Incubation with V(V)	Strain isolated from freshwater lake sediments	Zhang B. et al. (2025)
Reduced glutathione (GSH)		Thermophilic hydrogen-producing bacteria (*Bacilli* and *Clostridia*)	Spectrophotometric method—Ellman method (GSH quantification)	Comparing relative GSH levels between three different conditions: without V(V), with 50 mg/L V(V) and with 100 mg/L V(V)	Anaerobic sludge enriched for 60 days at 55 °C	Sample collected from anaerobic wastewater treatment station in Rizhao, Shandong (China)	Zheng et al. (2021)
		*Shewanella* sp. FDA-1	Quantification assay	Comparing enzyme activity with and without V(V)	Exposure to 610 mg/L V(V) for 1 day (24 h) at 30 °C		Cheng et al. (2025)*
		*Acidovorax* sp. BoFeN1	Spectrophotometric method - Ellman method (GSH quantification)	Comparing enzyme activity per protein with and without V(V)	Exposure to 10 mg/L V(V) for 5 days (120 h) at 30 °C	Strain isolated from Lake Constance coastal sediments	Fei et al. (2024)*
		*Tepidibacter mesophilus* VROV1	HPLC analysis (metabolite quantification)		Growth with 5 mM V(V) for 7.5 days (180 h) at 25 °C	Strain isolated from deep-sea sediments on the northern Central Indian Ridge	Kim et al. (2025)*
		*Thauera humireducens*	Spectrophotometric method—Ellman method (GSH quantification)	Comparing abundances with and without V(V)	Growth with 50 mg/L V(V) for 10 days at 30 °C	Strain provided by Fujian Agriculture and Forestry University (China)	Yan et al. (2025)*
Riboflavin		Thermophilic hydrogen-producing bacteria (*Bacilli* and *Clostridia*)	3DEEM fluorescence spectroscopy	Comparing the relative quenching of fluorescence	Anaerobic sludge enriched for 60 days at 55 °C	Sample collected from anaerobic wastewater treatment station in Rizhao, Shandong (China)	Zheng et al. (2021)
		*Pseudogulbenkiania* sp. 2002	Quantification enzyme assay	Comparing concentration with and without V(V)	Incubation with V(V)	Strain isolated from freshwater lake sediments	Zhang B. et al. (2025)*
		*Acidovorax* sp. BoFeN1	HPLC analysis (metabolite quantification) and quantification enzyme assay	Comparing concentration with and without V(V)	Exposure to 10 mg/L V(V) for 5 days (120 h) at 30 °C	Strain isolated from Lake Constance coastal sediments	Fei et al. (2024) and Zhang B. et al. (2025)*
Riboflavin synthase	*apbE*	*Enterococcus faecalis*	RT-qPCR (RNA analysis)	Comparing the gene expression under exposure to V: 1.7-fold change in expression with V(V)	Incubation with 250 mg/L V(V) in anaerobic conditions for 7 days at 30 °C	Strain isolated from V-contaminated soils	Zhang L. et al. (2025)
	*FOXRED1*	*Enterococcus faecalis*	RT-qPCR (RNA analysis)	Comparing the gene expression under exposure to V: 2.6-fold change in expression with V(V)	Incubation with 250 mg/L V(V) in anaerobic conditions for 7 days at 30 °C	Strain isolated from V-contaminated soils	Zhang L. et al. (2025)
	*ribF*	*Enterococcus faecalis*	RT-qPCR (RNA analysis)	Comparing the gene expression under exposure to V: 3.2-fold change in expression with V(V)	Incubation with 250 mg/L V(V) in anaerobic conditions for 7 days at 30 °C	Strain isolated from V-contaminated soils	Zhang L. et al. (2025)
	*ribBA*	*Shewanella* sp. FDA-1	RT-qPCR (RNA analysis)	Comparing the gene abundances obtained with and without V(V)	Exposure to 610 mg/L V(V) for 1 day (24 h) at 30 °C		Cheng et al. (2025)
	*ribD*	*Shewanella* sp. FDA-1	RT-qPCR (RNA analysis)	Comparing the gene abundances obtained with and without V(V)	Exposure to 610 mg/L V(V) for 1 day (24 h) at 30 °C		Cheng et al. (2025)
Sugar (maltose/G3P/polyamine/iron) ABC transporter substrate-binding protein		*Ochrobactrum tritici* 5bvl1	SDS-PAGE (analysis of protein profiles) and mass spectrometry (for protein identification)	Comparing protein profiles with and without V(V) and identifying the protein	Growth with 15 mM V(V) for 1 day (24 h) at 30 °C	Strain isolated from activated sludge in a Cr-contaminated area (Portugal)	Almeida et al. (2020)
Superoxide dismutase (SOD)		*Bacillus* sp. PFYN01	Quantification enzyme SOD kit	Comparing enzyme units per intact cell with and without V(V)	Incubation with 0 to 100 mg/L V(V) for 0.5 day (12 h) at 30 °C	Strain isolated from Majiatian V tailing reservoir in Panzhihua (China)	Zhou et al. (2022)*
		*Pseudogulbenkiania* sp. 2002	Quantification enzyme activity	Comparing enzyme units per cell with and without V(V)	Incubation with 10 mg/L V(V) in anaerobic conditions for 5 days (120 h) at 30 °C	Strain isolated from freshwater lake sediments	Fei et al. (2023)*
		*Acidovorax* sp. BoFeN1	Quantification enzyme activity	Comparing enzyme units per cell with and without V(V)	Incubation with 10 mg/L V(V) in anaerobic conditions for 5 days (120 h) at 30 °C	Strain isolated from lake coastal sediments	Fei et al. (2023)*
		Thermophilic hydrogen-producing bacteria (*Bacilli* and *Clostridia*)	Quantification enzyme	Comparing enzyme units per cell with and without V(V)	Anaerobic sludge enriched for 60 days at 55 °C	Sample collected from anaerobic wastewater treatment station in Rizhao, Shandong (China)	Zheng et al. (2021)
		*Enterococcus faecalis*	Quantification enzyme	Comparing enzyme units per cell with and without V(V)	Incubation with 250 mg/L V(V) in anaerobic conditions for 7 days at 30 °C	Strain isolated from V-contaminated soils	Zhang L. et al. (2025)*
		*Lactococcus raffinolactis*	Quantification enzyme activity	Comparing enzyme units per protein with and without V(V)	Exposure to V(V) for 10 days at 30 °C	Strain provided by Inner Mongolia Agricultural University (China)	Zhang B. et al. (2021)*
Thioredoxin reductase (TrxR)		*Shewanella* sp. FDA-1	Quantification activity kit	Comparing enzyme activity with and without V(V)	Exposure to 610 mg/L V(V) for 1 day (24 h) at 30 °C		Cheng et al. (2025)*
	*trxA*	*Bacillus megaterium* R01	RT-qPCR (RNA analysis)	Comparing the gene abundances obtained with and without V(V)	Exposure to different V(V) concentrations for 1 day (24 h) at 30 °C	Strain isolated from a stone coal mine region in Yiyang City, Hunan Province (China)	Huang et al. (2025)
	*trxR*	*Bacillus megaterium* R01	RT-qPCR (RNA analysis)	Comparing the gene abundances obtained with and without V(V)	Exposure to different V(V) concentrations for 1 day (24 h) at 30 °C	Strain isolated from a stone coal mine region in Yiyang City, Hunan Province (China)	Huang et al. (2025)
	*btuE*	*Shewanella* sp. FDA-1	RT-qPCR (RNA analysis)	Comparing the gene abundances obtained with and without V(V)	Exposure to 610 mg/L V(V) for 1 day (24 h) at 30 °C		Cheng et al. (2025)
Urea transport (ABC transporter)	*urtA*	*Pseudomonas balearica* 1A00204	Transcriptomic analysis (differential gene expression)	Comparing the gene expression with and without V(V)	Growth with 1 mM V(V) for 0.5 day (12 h) at 28 °C	Strain provided by the Marine Culture Collection of China (MCCC)	Gan et al. (2024)
	*urtC*	*Pseudomonas balearica* 1A00204	Transcriptomic analysis (differential gene expression)	Comparing the gene expression with and without V(V)	Growth with 1 mM V(V) for 0.5 day (12 h) at 28 °C	Strain provided by the Marine Culture Collection of China (MCCC)	Gan et al. (2024)
	*urtE*	*Pseudomonas balearica* 1A00204	Transcriptomic analysis (differential gene expression)	Comparing the gene expression with and without V(V)	Growth with 1 mM V(V) for 0.5 day (12 h) at 28 °C	Strain provided by the Marine Culture Collection of China (MCCC)	Gan et al. (2024)
V-nitrogenase	*vnfA*	*Azotobacter vinelandii*	RT-qPCR (RNA analysis)	Comparing the gene expression in strains with different mutations	Growth at 30 °C	Strains with different mutations and plasmids	Appia-Ayme et al. (2022)*
	*vnfZ*	*Azotobacter vinelandii*	RT-qPCR (RNA analysis)	Comparing the gene expression in strains with different mutations	Growth at 30 °C	Strains with different mutations and plasmids	Appia-Ayme et al. (2022)*
							

The revised articles marked with an asterisk (*) mention metabolic mechanisms to interact with V. FTIR, Fourier transform infrared spectroscopy; HPLC, high-performance liquid chromatography; RT-qPCR, reverse transcription quantitative polymerase chain reaction; SDS-PAGE, sodium dodecyl sulfate-polyacrylamide gel electrophoresis; 3DEEM, three-dimensional excitation emission matrix fluorescence spectroscopy.

In some bacteria, the ability to interact with or utilize V is linked to the presence of alternative nitrogenases. V-nitrogenases (such as, VnfK) mediate a connection between V and nitrogen fixation, using V as a cofactor along with other metals (such as molybdenum or iron) (Addo and Dos Santos, 2020; Lydon et al., 2020; Bellanger et al., 2024; Ratcliff et al., 2024). These enzymes catalyze the nitrogen reduction reaction:


$$N_{2}+14H^{+}+12e^{-}\to 2{{\mathrm{NH}}_{4}}^{+}+3H_{2}$$

and were reported to be present in various bacterial strains, such as *Azospirillum brasilense* Sp245 (Koul and Kochar, 2021), *Rhodopseudomonas palustris* CGA766 (Luxem et al., 2020a), *Holophaga* sp. (Reji and Zhang, 2022), *Methylocystis heyeri* H2^T^ (Oshkin et al., 2020) *Methanosarcina acetivorans* (Chanderban et al., 2023), *Clostridium pasteurianum* (Berthomieu et al., 2022)*, Rhodopseudomonas palustris* CGA009 (Luxem et al., 2020b), and *Azotobacter vinelandii* (Pence et al., 2021). Particularly, an *Azotobacter vinelandii* strain was mutated, and the gene abundances were compared between the mutated strains (Appia-Ayme et al., 2022). Exploring RT-qPCR techniques, two genes encoding V-nitrogenases presented more abundance with V(V): *vnfA* and *vnfZ* (Appia-Ayme et al., 2022).

The relationship between nitrogen metabolism and V is not restricted to V-nitrogenases since recent studies have linked the presence of bacterial denitrification-related genes to the enzymatic processes involved in the reduction of V(V) to V(IV) (Sun et al., 2025). This link may be explained by the molecular similarity between V(V) and nitrate structures (Yan et al., 2023). Nitrite reductases were reported to promote V(V)-bioreduction, and functional genes coding for nitrite reductases, *nirB, nirD, nirK, nrfA* and *nirS* have been identified in many bacterial strains from different genera, such as *Acidovorax* (Fei et al., 2023, 2024)*, Pseudomonas* (Gan et al., 2024)*, Shewanella* (Cheng et al., 2025)*, Pseudogulkiania* (Fei et al., 2023), and *Lactococcus* (Zhang B. et al., 2021). Nitrate reductase genes were also described as key genes in the V(V)-bioreduction process. Some examples are the *narG* gene in genera *Acidovorax* (Fei et al., 2024)*, Lactococcus* (Zhang B. et al., 2021), and *Polaromonas* (Sun et al., 2020) and the *napA* gene in *Lactococcus* (Zhang B. et al., 2021)*, Acidovorax* (Fei et al., 2024), and *Pseudogulbenkiania* (Fei et al., 2023). Nitric and nitrous oxidoreductase enzymes (NOR and NOS) were also reported to be related to V-resistance in some bacteria, such as *Acidovorax* sp. BoFeN1 (Fei et al., 2024) and *Pseudogulbenkiania* sp. 2002 (Fei et al., 2023). The majority of these studies correlate V(V)-exposure with denitrification genes by differences in gene abundances, but some articles used quantification methods to evaluate the enzymatic activity of nitrate reductase (NAP), nitrite reductase (NIR), nitric oxidoreductase (NOR) and nitrous oxidoreductase (NOS), which confirms this relationship, particularly for *Bacillus* sp. PFYN01 (Zhou et al., 2022), *Acidovorax* sp. BoFeN1 (Fei et al., 2023, 2024), and *Pseudogulbenkiania* sp. 2002 (Fei et al., 2023). Some articles also reported the existence of these denitrification genes in microbial communities rather than in isolated strains (He et al., 2021, 2023; Wang et al., 2021; Liu et al., 2023; Yan et al., 2023; Sun et al., 2025).

Microbes can protect themselves from toxic V(V) by using membrane proteins encoded by metal resistance genes (Yan et al., 2023). Chromate (Cr(VI)) resistance in bacteria is linked to an efflux system that facilitates the movement of Cr(VI) from inside to outside bacterial cells. This efflux system is comprised of the *chr* operon, which includes several genes, such as *chrA* and *chrC*. The first gene, *chrA,* encodes the Cr(VI) efflux transporter, known as ChrA. The second gene, *chrC*, encodes a putative superoxide dismutase (SOD) (Branco et al., 2008). Some studies describe the Chr system to have Cr(VI)-reductase functions, promoting the reduction of Cr(VI) to Cr(III). This reductase function requires NADPH, which enhances the production of reactive oxygen species. Consequently, this activates enzymes that cope with oxidative stress, such as SOD and catalase (Ramli et al., 2023). Research has already indicated an association between the presence of genes *chrA* and *chrC* and V(V)-exposure, suggesting a potential relationship between V(V) and Cr(VI) resistance mechanisms (Shi et al., 2020; Yan et al., 2023). This relationship may be attributed to the fact that V and Cr are neighboring elements in the periodic table, both belonging to the 4th period.

However, it is possible to conclude from the literature that the relationship between V and other metals is not restricted solely to Cr(VI) (Shi et al., 2020). *The smtB* gene has been linked to V-resistance in an *Anaeromyxobacter* sp. strain (Yan et al., 2023). This gene is part of the ArsR-SmtB family, which consists of metalloregulatory proteins. It functions as a repressor of the *smtA* gene and can bind with zinc (Zn(II)), as well as cadmium (Cd(II)). Although it is not known to bind to arsenate (As(V)) or arsenite (As(III)), it shares a family with ArsR, a regulator of the *ars* operon responsible for As-resistance (Turner et al., 1996). Additionally, the *czcD* gene has been identified as being related to V-resistance in microorganisms and potentially with V-bioaccumulation (Fierros-Romero et al., 2020; Yan et al., 2023). This gene has been identified in *Microbacterium liquefaciens* MNSH1-9K-1 (Fierros-Romero et al., 2020) and in a microbial community (particularly in strains *Anaeromyxobacter* sp. bin.29 and *UBA6901* sp. bin.68) (Yan et al., 2023). The Czc complex is a transporter for cobalt (Co(II)), Zn(II) and Cd(II). It acts as a cation diffusion facilitator, transporting Cd(II), Co(II), Zn(II) and Ni(II), and may also play a role in V-resistance (Fierros-Romero et al., 2020).

The bioreduction of V(V) has been linked to a multicomponent electron transport chain present in certain bacteria (Shi et al., 2020). In these bacteria, the proteins of this electron transport chain are located on the bacterial membrane and are responsible for synthesizing electron shuttles, specifically molecules of cytochrome c (He et al., 2021, 2023; Wang et al., 2021; Fei et al., 2024; Zhang B. et al., 2025). The multicomponent electron transport includes CymA (cytoplasmic membrane component) (Sun et al., 2020; Yan et al., 2023), MtrA, MtrB, and MtrC (membrane cytochrome) from c-type cytochromes (Shi et al., 2020; Yan et al., 2023), CcmC (cytochrome c maturation protein) (Yan et al., 2023), as well as OmcA and OmcB (outer membrane cytochrome) (Shi et al., 2020; Sun et al., 2020; He et al., 2021; Yan et al., 2023). Cytochrome c molecules transfer electrons to denitrifying enzymes, which promote denitrification and V(V)-bioreduction (Fei et al., 2024). This process is facilitated by bacteria, such as *Acidovorax* sp. BoFeN1 (Fei et al., 2024; Zhang B. et al., 2025), *Pseudogulbenkiania* sp. 2002 (Zhang B. et al., 2025), and *Polaromonas* spp. (Sun et al., 2020), that extracellularly transfer electrons from microorganisms to multivalent metal ions, in this case V(V). V-reducing bacteria have been shown to promote V(V)-bioreduction by binding V with reductases of other electron acceptors for detoxification.

Furthermore, a study demonstrated the overexpression in protein profiles of a methionine ABC transporter and a sugar (maltose/G3P/polyamine/iron) ABC transporter substrate-binding protein in *Ochrobactrum tritici* strains when exposed to 15 mM V(V) (Almeida et al., 2020). Methionine ABC transporter was already associated with metal ion homeostasis and oxidative stress response processes (Zhang et al., 2012). The sugar ABC transporter (NCBI Accession number WP_003091922.1) is described as responsible for the energy coupling in an ABC transporter complex involved in the import of inorganic ions (Almeida et al., 2020). Additionally, another study identified a different transporter associated with V(V)-exposure. The strain *Pseudomonas balearica* 1A00204 exhibited increased gene expression levels for the *urtA*, *urtC*, and *urtE* genes in response to V(V)-exposure. These genes encode the urea transporter Urt (Gan et al., 2024). The mentioned studies suggest a connection between the response to V(V) and energy metabolism, proposing that these ABC transporters may be involved in the bidirectional movement of V across the cell membrane (Almeida et al., 2020; Gan et al., 2024).

Low-molecular-weight thiols, which are substances rich in -SH groups, have been shown to play a role in V-resistance. These substances include different molecules, such as reduced glutathione (GSH) (Zheng et al., 2021; Fei et al., 2024; Cheng et al., 2025; Kim et al., 2025; Yan et al., 2025), bacillithiol (BSH) (Huang et al., 2025), cysteine (Cys) (Huang et al., 2025), and thioredoxin reductase (TrxR) (Cheng et al., 2025; Huang et al., 2025). Notably, these molecules differ in structure, and not all are present in every bacterial strain. For example, BSH is typically found in certain Gram-positive bacterial strains, particularly in *Bacillus* and *Staphylococcus* genera, while GSH is primarily present in Gram-negative bacteria and is generally absent in Gram-positive strains (Fei et al., 2024; Cheng et al., 2025; Huang et al., 2025; Kim et al., 2025; Yan et al., 2025). Although these molecules have distinct structures, sulfate-reducing bacteria can facilitate the electron transfer from these terminal electron donors in the electron transport chain. The bacteria use this -SH group-containing substances to form disulfides (RS-SR) and bioreduce V(V) to V(IV) (Huang et al., 2025). Additionally, nicotinamide adenine dinucleotide (NADH)-dependent reductase, an intracellular coenzyme, has also been identified as an important electron donor in the cytoplasmic bioreduction of V(V) (He et al., 2021, 2023; Wang et al., 2021; Zhang B. et al., 2021; Zhang L. et al., 2025; Zheng et al., 2021; Fei et al., 2024; Yan et al., 2025). NADH serves as a major electron donor for denitrification and V(V)-bioreduction (Fei et al., 2024). Other proteins associated with electron transport, such as riboflavin and quinones, have also been detected in studies involving exposure to V(V) (Zheng et al., 2021; Fei et al., 2024; Cheng et al., 2025; Zhang B. et al., 2021; Zhang L. et al., 2025). The detection of various proteins and molecules involved in electron transfer highlights the significance of electron donors, suggesting that V(V) acts as an electron acceptor.

Similar to the effects of other metals, such as Cr(VI), exposure to V appears to induce oxidative stress, requiring the involvement of additional detoxification processes mediated by enzymes (Ramli et al., 2023; Zhang B. et al., 2021). The V(V)-bioreduction process can lead to the production of intracellular reactive oxygen species (ROS), which enhance many stress protection mechanisms, such as SOD and catalase, during V-exposure (Zhang B. et al., 2021; Zhang L. et al., 2025; Zheng et al., 2021; Zhou et al., 2022; Fei et al., 2023). Despite the increased levels of intracellular ROS during V(V)-bioreduction, the antioxidant activities of SOD and catalase help mitigate the effects of ROS, preserving cellular integrity (Zhang B. et al., 2021; Zhang L. et al., 2025).

From an extracellular perspective, extracellular polymeric substances (EPS) are bacterial polymers that play an important role in mediating microbial interactions with V(V) (Wang et al., 2021; Zheng et al., 2021). EPS, produced by many bacteria, can enhance both V(V)-bioreduction and nitrogen metabolism (Sun et al., 2025). EPS from denitrifying strains, particularly from the genera *Bacillus* (Zhou et al., 2022; Yan et al., 2025) and *Thauera* (Yan et al., 2025), have been shown to chemically react with V(V). This interaction potentially allows the binding of the metal and serves as an extracellular electron donor, thereby facilitating V(V)-bioreduction (Zhou et al., 2022; Yan et al., 2025). Additionally, several strains have been identified as capable of bioreducing V(V) through EPS secretion, including *Acidovorax* sp. BoFeN1 (Fei et al., 2024), *Pseudogulbenkiania* sp. 2002 (Zhang B. et al., 2025), *Bacillus* sp. PFYN01 (Zhou et al., 2022), *Bacillus subtilis* (Yan et al., 2025)*, Thauera humireducens* (Yan et al., 2025)*, Shewanella* sp. FDA-1 (Cheng et al., 2025), and *Enterococcus faecalis* (Zhang L. et al., 2025). The strains *Acidovorax* sp. BoFeN1 (Fei et al., 2024), *Pseudogulbenkiania* sp. 2002 (Zhang B. et al., 2025), and *Enterococcus faecalis* (Zhang L. et al., 2025) were also shown to synthesize riboflavin. This synthesis was detected through either increased riboflavin activity or the expression of riboflavin synthase genes. Riboflavin and quinones were reported to form complexes with V(V) and be secreted to accelerate extracellular electron transfer (Zhang B. et al., 2025; Zhang L. et al., 2025).

## 5 Applications of vanadium biomobilization

The methods applied nowadays for removing heavy metals from waste are diverse, e.g., chemical precipitation, coagulation-flocculation, membrane filtration, and adsorption. However, these physicochemical methods may be more costly and less effective in the treatment of wastes with lower metal concentrations compared to biological methods (Samaei et al., 2022). In recent years, microorganisms have been the focus of many studies to develop new biotechnologies to recycle heavy metals from metal-contaminated environments, exploring their capacity to accumulate, leach or reduce heavy metals (Caldeira et al., 2021a; Tang et al., 2023). For example, there are already published works documenting microorganisms that accumulate heavy metals from solutions, such as indium and tellurium (Caldeira et al., 2021b; Farias et al., 2022). In addition to these metals, several studies have also investigated the ability of microorganisms to remove V from either solutions or residues containing V (Almeida et al., 2020; Bellenberg et al., 2021). This has been achieved using specific strategies for: V-bioaccumulation, increasing V-concentration in microorganisms; V-bioleaching, converting V from an insoluble to a soluble form; V(V)-bioreduction, reducing V(V) to a lower oxidation state; and removal of V, decreasing V-concentration in the medium (Table 2). A variety of conditions have been tested, including different bacterial strains (both isolated strains and microbial communities), initial V-concentrations, and various liquid and growth conditions, ranging from anaerobic to aerobic environments. The incubation times varied from hours to days, and factors such as pH and electron donors were also considered, along with quantification methods. However, some articles describe results without including necessary controls. For example, some discuss V(V)-bioreduction using only methods for V(V) quantification, whereas total V quantification methods, as well as methods for analyzing the V valence state, should have been used. Additionally, metal precipitation artifacts were identified in many articles; some of these articles analyzed the metal precipitates and found reduced forms of V.

**Table 2:** List of processes exploring bacteria’s ability for V-biomobilization and values of V obtained, from information provided in reported studies.

Bacterial strains	V concentration		Tested conditions							Quantification method	Reference
	Initial	Final/efficiency (%)	Liquid	Growth conditions	Time	pH	Biomass	Electron	Relevant information		
Bioaccumulation											
*Vibrio* sp. CD2-102	92 mg/L V(V)	4,905 μg/g dry weight	Assay buffer (0.5 M NaCl and 0.05 mM sodium phosphate)	25 °C, 180 rpm	1 day (24 h)	3	Cells in the log phase; growth in standard medium (yeast extract 2.5 g/L, peptone 5.0 g/L, and glucose 1.0 g/L)	Electron acceptor: V(V); electron donor: glucose	Bacterial strains were isolated from *Ciona robusta*	AAS (for total V quantification)	Yuliani et al. (2024)
		2,150 μg/g dry weight				5					
		9 μg/g dry weight				7					
*Pseudoalteromonas* sp. CD2-88	92 mg/L V(V)	4,342 μg/g dry weight	Assay buffer	25 °C, 180 rpm	1 day (24 h)	3	Cells in the log phase; growth in standard medium	Electron acceptor: V(V); electron donor: glucose	Bacterial strains were isolated from *Ciona robusta*	AAS (for total V quantification)	Yuliani et al. (2024)
		1,132 μg/g dry weight				5					
		25 μg/g dry weight				7					
*Ochrobactrum tritici* 5bvl1	552 mg/L V(V)	1.3 μg V/mg total protein	Reasoner’s 2A broth (casein acid hydrolysate 0.5 g/L, yeast extract 0.5 g/L, proteose peptone 0.5 g/L, dextrose 0.5 g/L, starch 0.5 g/L, dipotassium phosphate 0.3 g/L, magnesium phosphate 0.024 g/L, sodium pyruvate 0.3 g/L)	30 °C, shaking	0.125 day (3 h)	7.2	OD_600_ = 0.7	Electron acceptor: V(V); electron donor: carbon source	Control: bacteria without V(V)	ICP-MS (for total V quantification)	Almeida et al. (2020)
*Ochrobactrum tritici* SCII24^T^		3.2 μg V/mg total protein									
*Ochrobactrum tritici* 5bvl1	2,759 mg/L V(V)	3.5 μg V/mg total protein									
*Ochrobactrum tritici* SCII24^T^		8.5 μg V/mg total protein									
*Ochrobactrum tritici* 5bvl1	552 mg/L V(V)	1.2 μg V/mg total protein	Reasoner’s 2A broth supplemented with Cr(VI) 0.02 g/L	30 °C, shaking	0.125 day (3 h)	7.2	OD_600_ = 0.7	Electron acceptor: V(V); electron donor: carbon source	Control: bacteria without V(V)	ICP-MS (for total V quantification)	Almeida et al. (2020)
*Ochrobactrum tritici* SCII24^T^		3.5 μg V/mg total protein									
*Ochrobactrum tritici* E117		1.5 μg V/mg total protein									
*Ochrobactrum tritici* E117:*chrA*		1.2 μg V/mg total protein									
*Ochrobactrum tritici* 5bvl1	2,759 mg/L V(V)	5 μg V/mg total protein	Reasoner’s 2A broth supplemented with Cr(VI) 0.02 g/L	30 °C, shaking	0.125 day (3 h)	7.2	OD_600_ = 0.7	Electron acceptor: V(V); electron donor: carbon source	Control: bacteria without V(V)	ICP-MS (for total V quantification)	Almeida et al. (2020)
*Ochrobactrum tritici* SCII24^T^		10 μg V/mg total protein									
*Ochrobactrum tritici* E117		5 μg V/mg total protein									
*Ochrobactrum tritici* E117:*chrA*		3.5 μg V/mg total protein									
*Vibrio* sp. CD2-102	92 mg/L V(IV)	142 μg/g dry weight	Assay buffer	25 °C, 180 rpm	1 day (24 h)	3	Cells in the log phase; growth in standard medium	Electron acceptor: V(V); electron donor: glucose	Bacterial strains were isolated from *Ciona robusta*	AAS (for total V quantification)	Yuliani et al. (2024)
		817 μg/g dry weight				5					
*Pseudoalteromonas* sp. CD2-88	92 mg/L V(IV)	188 μg/g dry weight	Assay buffer	25 °C, 180 rpm	1 day (24 h)	3	Cells in the log phase; growth in Standard medium	Electron acceptor: V(V); electron donor: glucose	Bacterial strains were isolated from *Ciona robusta*	AAS (for total V quantification)	Yuliani et al. (2024)
		276 μg/g dry weight				5					
Bioleaching											
*Acidithiobacillus thiooxidans* (DSM 9463)	438 mg/L (5.50% in SDC sample)	6.4 mg/L V/98.5% leached (0.22% in SDC sample)	AT medium (ammonium sulfate 2 g/L, magnesium sulfate 0.25 g/L, dipotassium phosphate 0.1 g/L, potassium chloride 0.1 g/L, and elemental sulfur (S(0)) 10 g/L)	150 rpm I—room temperature II—room temperature III—85 °C IV—130 °C	I—0.7 day (16 h) II—0.7 day (16 h) III—0.8 day (2 h + 16 h) I—0.1 day (2 h)	2.5 I—3 II—2 III—2	1 mL of inoculum was mixed with SDC sample, 50 mL of AT medium and S(0) 20 g/L	Electron acceptor: V(V); electron donor: S(0)	Bacterial strain provided by Leibniz-Institut DSMZ; spent desulfurization catalyst (SDC)	ICP-MS (for total V quantification)	Mikoda et al. (2020)
Mutagenized *Bacillus mucilaginosus*	0.75% V in shale sample	61.2% leached	Bacterial basal medium (BBM) (sucrose 5 g/L, disodium phosphate 2.0 g/L, calcium carbonate 0.1 g/L, magnesium sulfate 0.5 g/L, and iron(III) chloride 0.005 g/L)	30 °C, 220 rpm	35 days	6.0	Cells in the log phase; 10% (v/v) inoculum in 100 mL medium	Electron acceptor: V(V); electron donor: carbon source	During the experiment, different values of pH were tested with a shale sample containing 0.75% V	Titration: ferrous sulfate method	Tian et al. (2023)
		64.9% leached				6.5					
		67.3% leached				7.0					
		68.5% leached				7.5					
		68.0% leached				8.0					
		58.8% leached				8.5					
		53.9% leached				9.0					
Mutagenized *Bacillus mucilaginosus*	0.75% V in shale sample	69% leached	BBM with ammonium nitrate 0.5 g/L	30 °C, 220 rpm	35 days	7.5	Cells in the log phase; 10% (v/v) inoculum in 100 mL medium	Electron acceptors: V(V), and nitrate; electron donor: carbon source	During the experiment, different concentrations of ammonium nitrate were tested with a shale sample containing 0.75% V	Titration: ferrous sulfate method	Tian et al. (2023)
		58% leached	BBM with ammonium nitrate 1 g/L								
		58% leached	BBM with ammonium nitrate 2 g/L								
		56% leached	BBM with ammonium nitrate 3 g/L								
		63% leached	BBM with ammonium nitrate 4 g/L								
Mutagenized *Bacillus mucilaginosus*	15.4% V in stone coal	25% leached (11.6% V in stoal)	Culture medium (disodium phosphate 3.0 g/L, ammonium sulfate 2.0 g/L, magnesium sulfate 0.5 g/L, potassium chloride 0.1 g/L, calcium carbonate0.1 g/L, and iron chloride 0.01 g/L) with 10 g/L stone coal and 20 g/L sucrose	30 °C, 180 rpm	20 days	7.0	1 mL of inoculum and stone coal 10 g/L	Electron acceptor: V(V); electron donor: stone coal	Stone coal from a V ore Different conditions were tested	ICP-MS (for total V quantification) and XRF (for V content in stone coal)	Dong et al. (2024)
*Pseudomonas* sp. LM27	1,500 mg/kg	17% leached	Modified mineral salts medium (dipotassium phosphate 0.8 g/L and monopotassium phosphate 0.2 g/L) supplemented with 10 mM glucose	22 °C, static aerobic and dark conditions	30 days	7.0	1.5 × 10^6^ cells/mL	Electron acceptor: V(V); electron donor: glucose	Control: sterile control	AAS (for total V quantification)	Stasiuk and Matlakowska (2021)
Microbial community (e.g., *Acidovorax, Delftia* and *Pseudomonas*)	0.46 mg/L V (0.23% V in stone coal)	0.66 mg/L V/144% leached	Bioreactor medium (acetate 2.05 g/L, monopotassium phosphate 0.07 g/L, and ammonium chloride 0.31 g/L)	22 °C, bioreactor with constant airflow (6.0 mL/min), for 6 consecutive operating cycles	3 days	7.0	5 mL of aerobiotic sludge and 95 mL of Bioreactor medium	Electron acceptor: V(V); electron donor: carbon source (from the stone coal)	Controls: (1) anaerobic sludge, and (2) abiotic control	ICP-MS (for total V quantification), and XPS (for V valence state analysis)	Zhang et al. (2022)
		0.68 mg/L V/148% leached			6 days						
		0.71 mg/L V/154% leached			9 days						
		0.22 mg/L V/48% leached			12 days						
		0.11 mg/L V/24% leached			15 days						
		0.1 mg/L V/22% leached			18 days						
Bioreduction											
*Vibrio* sp. CD2-102	0.5 mM V(V)	0.497 mM V(IV)/99.7% reduced	Standard medium (yeast extract 2.5 g/L, peptone 5.0 g/L, and glucose 1.0 g/L)	25 °C, 180 rpm	0.5 day (overnight)	ND	Cells in the log phase; growth in standard medium	Electron acceptor: V(V); electron donor: glucose	Bacterial strains were isolated from *Ciona robusta*	IC (for V valence state analysis)	Yuliani et al. (2024)
*Pseudoalteromonas* sp. CD2-88		0.4985 mM V(IV)/99.4% reduced									
*Thauera humireducens*	50 mg/L V(V)	18.7 mg/L V(IV) (precipitate)/37.3% reduced	Aqueous solution (calcium chloride 0.25 g/L, magnesium chloride 1.06 g/L, sodium chloride 0.45 g/L, potassium chloride 0.03 g/L, sodium bicarbonate 0.81 g/L, ammonium chloride 0.16 g, and monopotassium phosphate 0.03 g/L) with 0.3 g/L ethanol	30 °C, 135 rpm	10 days	7.0	OD_600_ = 0.50 Cells from growth in Luria–Bertani medium (yeast extract 5 g/L, tryptone 10 g/L, and sodium chloride 10 g/L) for 24 h	Electron acceptor: V(V); electron donor: ethanol	Control: sterilized biomass	Spectrophotometric method (for V(V) quantification), and XPS (for V valence state analysis)	Yan et al. (2025)
*Bacillus subtilis*	50 mg/L V(V)	19 mg/L V(IV) (precipitate)/38% reduced	Aqueous solution with 0.3 g/L ethanol	30 °C, 135 rpm	10 days	7.0	OD_600_ = 0.50 Cells from growth in Luria–Bertani medium for 24 h	Electron acceptor: V(V); electron donor: ethanol	Control: sterilized biomass	Spectrophotometric method (for V(V) quantification), and XPS (for V valence state analysis)	Yan et al. (2025)
*Tepidibacter mesophilus* VROV1	548.7 mg/L V(V)	365.8 mg/L V(IV) (precipitate)/67% reduced	Carbonate-buffered medium (sodium chloride 21 g/L, sodium bicarbonate 9 g/L, ammonium chloride 1 g/L, monopotassium phosphate 0.5 g/L, magnesium chloride 1.4 g/L, calcium chloride 0.11 g/L, sodium citrate 0.3 g/L, yeast extract 1 g/L, sodium lactate solution (60% w/w) 0.7 mL/L, and trace element solution)	25 °C, anaerobic conditions	7.5 days (180 h)	7.4	ND	Electron acceptors: V(V), nitrate and SO₄^2−^; electron donor: glucose	Control: abiotic	ICP–AES (for total V quantification), spectrophotometric method (for V(V) quantification), and XPS (for V valence state analysis)	Kim et al. (2025)
		182.9 mg/L V (dissolved)/33%									
*Lactococcus raffinolactis*	50 mg/L V(V)	V(IV) (precipitate)/86.5% reduced	Aqueous solution (calcium chloride 0.25 g/L, Magnesium chloride 1.06 g/L, sodium chloride 0.45 g/L, potassium chloride 0.03 g/L, sodium bicarbonate 0.81 g/L, ammonium chloride 0.16 g/L, and monopotassium phosphate 0.03 g/L)	30 °C	10 days	7.0	OD_600_ = 0.50	Electron acceptors: V(V) and nitrate; electron donor: citrate (from the sediment)	Sediment from V tailing with pH 8 and 0.34 mg V/kg; Controls: (1) sole citrate, (2) sole microbe, and (3) sterilized biomass	ICP-MS (for total V quantification), and XPS (for V valence state analysis)	Zhang B. et al. (2021)
		6.75 mg/L V(V)									
	25 mg/L V(V)	5 mg/L V(V)/80% reduced									
	50 mg/L V(V)	11 mg/L V(V)/78% reduced				5.0					
		10 mg/L V(V)/80% reduced				6.0					
		6 mg/L V(V)/88% reduced				7.0					
		9 mg/L V(V)/82% reduced				8.0					
	75 mg/L V(V)	15 mg/L V(V)/80% reduced				7.0					
	100 mg/L V(V)	21 mg/L V(V)/79% reduced									
Microbial community (e.g., *Thiobacillus, Petrimonas, Mesotoga,* and *Longilinea*)	10 mg/L V(V)	0 mg/L V(V)/100% reduced to V(IV)	Synthetic V(V)-contaminated groundwater (calcium chloride 0.25 g/L, ammonium chloride 0.04 g/L, magnesium chloride 1.06 g/L, sodium chloride 0.45 g/L, potassium chloride 0.03 g/L, and monopotassium phosphate 0.03 g/L)	22 °C, column with flow rate of 0.125 mL/min	40 days	7	50 mL of anaerobic consortium from brewery wastewater treatment	Electron acceptor: V(V); electron donor: S(0) (from mackinawite)	Control: abiotic column	ICP–MS (for total V quantification), spectrophotometric method (for V(V) quantification), and XPS (for V valence state analysis)	He et al. (2021)
	10 mg/L V(V)	1.6 mg/L V(V)/84.4% reduced to V(IV)		22 °C, column with flow rate of 0.250 mL/min	20 days (41–60 days)	7					
	50 mg/L V(V)	5.2 mg/L V(V)/89.6% reduced to V(IV)		22 °C, column with flow rate of 0.125 mL/min	30 days (61–90 days)	7.5					
	50 mg/L V(V)	22.2 mg/L V(V)/55.6% reduced to V(IV)	Synthetic V(V)-contaminated groundwater with nitrate 0.05 g/L	22 °C, column with flow rate of 0.125 mL/min	30 days (91–120 days)	7.8		Electron acceptors: V(V) and nitrate; electron donor: S(0) (from mackinawite)			
	50 mg/L V(V)	5.1 mg/L V(V)/89.8% reduced to V(IV)	Synthetic V(V)-contaminated groundwater	22 °C, column with flow rate of 0.125 mL/min	30 days (121–150 days)	7.5		Electron acceptor: V(V); electron donor: S(0) (from mackinawite)			
Microbial community (e.g., *Azospira, Luteolibacter, Azotobacter, Meniscus, Pseudomonas, Thiobacillus,* and *Bacteroides*)	10 mg/L V(V)	V(IV) (precipitate)	Freshwater environment medium (calcium chloride 0.25 g/L, magnesium chloride 1.06 g/L, potassium phosphate 0.03 g/L, sodium bicarbonate 0.81 g/L, ammonium chloride 0.16 g/L, potassium chloride 0.03 g/L, and sodium chloride 0.45 g/L) with U(VI) 0.01 g/L	Anaerobic continuous column	140 days	7.5	50 mL sludge (inoculum) with 100 g woodchips and 100 g S(0)	Electron acceptors: V(V) and U(VI); electron donor: S(0) and woodchips	Sludge from an aquifer near V-tailings pond of V-mine in Panzhihua (China)	ICP–MS (for total V quantification), spectrophotometric method (for V(V) quantification), and XPS (for V valence state analysis)	Liu et al. (2022)
Microbial community (*Deltaproteobacteria, Anaerolineae, Campylobacteria, Bacteroidia* and *Gammaproteobacteria*)	10 mg/L V(V)	V(IV) (precipitate)	Synthetic groundwater solution (ammonium chloride 0.16 g/L, calcium chloride 0.25 g/L, magnesium chloride 1.06 g/L, sodium chloride 0.45 g/L, potassium chloride 0.03 g/L, sodium bicarbonate 0.36 g/L, and monopotassium phosphate 0.03 g/L) with Cr(VI) 10 mg/L and organic carbon source	22 °C, batch	3 days (72 h)	ND	Anaerobic microbial community from brewery wastewater treatment (China)	Electron acceptors: V(V) and Cr(VI); electron donor: S(0)	Inoculum mixed with S(0)	XPS (for V valence state analysis)	Shi et al. (2020)
Microbial community (e.g., *Bacilli* and *Clostridia*)	100 mg/L V(V)	2.3 mg/L V(V)/97.75% reduced to V(IV)	Deionized water with glucose 10 g/L	55 °C, anaerobic hydrogen fermenter	5 days (120 h)	5.8	10% of anaerobic sludge (sludge was heated at 120 °C for 30 min and then enriched at 55 °C for 60 days)	Electron acceptor: V(V); electron donor: glucose	Control: without V(V)	Spectrophotometric method (for V(V) quantification), and XPS (for V valence state analysis)	Zheng et al. (2021)
	100 mg/L V(V)	2.5 mg/L V(V)/97.5% reduced to V(IV)			8.1 days (195 h)						
	100 mg/L V(V)	5 mg/L V(V)/95% reduced to V(IV)			7.3 days (175 h)						
*Shewanella* sp. FDA-1	122 mg/L V(V)	55 mg/L V(IV)/45% reduced to V(IV)	Modified mineral salt medium (MSM) (calcium chloride 0.25 g/L, magnesium chloride 1.06 g/L, sodium chloride 30 g/L, potassium chloride 0.03 g/L, sodium bicarbonate 0.81 g/L, ammonium chloride 0.16 g/L, potassium phosphate 0.03 g/L) with lactate 2 g/L	30 °C, anaerobic conditins	0.3 day (8 h)	7.2	OD_600_ = 0.2	Electron acceptor: V(V); electron donor: lactate	FDA-1 strain grown in modified Luria–Bertan medium (peptides 5 g/L, yeast extract 10 g/L, sodium chloride 20 g/L), 150 rpm, until OD_600_ = 2.0	Spectrophotometric method (for total V, V(V) and V(IV) quantifications)	Cheng et al. (2025)
		61 mg/L V(V)									
	244 mg/L V(V)	104 mg/L V(IV)/43% reduced to V(IV)									
		73 mg/L V(V)									
	610 mg/L V(V)	214 mg/L V(IV)/35% reduced to V(IV)									
		171 mg/L V(V)									
*Polaromonas* spp.	68 mg/L V(V)	V(IV) (precipitate)/93% reduced	Mineral salt medium (monopotassium phosphate 1.5 g/L, disodium phosphate 7.9 g/L, ammonium chloride 0.3 g/L, magnesium chloride hexahydrate 0.1 g/L, trace element solution 1 mL/L, and vitamin solution 1 mL/L) with 6 mM acetate	2nd anaerobic enrichment	49 days	3.0 (tailing paste)	Set of anaerobic enrichment cultures + 4% tailings	Electron acceptor: V(V); electron donor: acetate	Controls: (1) killed control (autoclaved tailings), and (2) without V(V) (no-electron acceptor)	ICP-OES (for total V quantification), spectrophotometric method (for V(V) quantification) and XPS (for V valence state analysis)	Sun et al. (2020)
		5 mg/L V(V)									
	60 mg/L V(V)	V(IV) (precipitate)/92% reduced		4th anaerobic enrichment	31 days						
		5 mg/L V(V)									
Removal											
*Acidovorax* sp. BoFeN1	10 mg/L V(V)	1 mg/L V/90% removed	Synthetic groundwater solution (calcium chloride 0.25 g/L, magnesium chloride 1.06 g/L, sodium chloride 0.45 g/L, potassium chloride 0.03 g/L, sodium bicarbonate 0.81 g/L, monopotassium phosphate 0.03 g/L, and sodium acetate 0.21 g/L)	30 °C, anaerobic conditions	5 days (120 h)	ND	5.5–6.5 × 10^7^ cells/mL	Electron acceptor: V(V); electron donor: sodium acetate	Controls: (1) control without contaminants, (2) sterile control, and (3) a bacterial inactivation control	ICP-MS (for total V quantification)	Fei et al. (2024)
		2.35 mg/L V/76.5% removed	Synthetic groundwater solution with 0.01 g/L nitrate					Electron acceptors: V(V) and nitrate; electron donor: sodium acetate			
		4 mg/L V/60% removed				6.0					
		2 mg/L V/80% removed				7.0					
		3.5 mg/L V/65% removed				8.0					
*Acidovorax* sp. BoFeN1	10 mg/L V(V)	1 mg/L V/89.0% removed	Synthetic groundwater solution (calcium chloride 0.25 g/L, magnesium chloride hexahydrate 1.06 g/L, sodium chloride 0.45 g/L, potassium chloride 0.03 g/L, sodium bicarbonate 0.81 g/L, potassium dihydrogen phosphate 0.03 g/L, sodium acetate 1.03 g/L)	30 °C, anaerobic conditions	5 days (120 h)	7.0	5.5–6.5 × 10^7^ cells/mL (OD_600_ = 1.0)	Electron acceptor: V(V); electron donor: sodium acetate	Control: without V(V) addition	ICP-MS (for total V quantification)	Fei et al. (2023)
	50 mg/L V(V)	10 mg/L V/80% removed									
	100 mg/L V(V)	40 mg/L V/60% removed									
*Pseudogulbenkiania* sp. 2002	10 mg/L V(V)	0.4 mg/L V/96% removed	Synthetic groundwater solution	30 °C, anaerobic conditions	5 days (120 h)	7.0	5.5–6.5 × 10^7^ cells/mL (OD_600_ = 1.0)	Electron acceptor: V(V); electron donor: acetate	Control: without V(V) addition	ICP-MS (for total V quantification)	Fei et al. (2023)
	50 mg/L V(V)	18 mg/L V/64% removed									
	100 mg/L V(V)	55 mg/L V/45% removed									
Microbial community (e.g., *Azospira, Luteolibacter, Azotobacter, Meniscus, Pseudomonas, Thiobacillus* and *Bacteroides*)	10 mg/L V(V)	0.05 mg/L V(V)/99.5% removed	Freshwater environment medium (calcium chloride 0.25 g/L, magnesium chloride 1.06 g/L, potassium phosphate 0.03 g/L, sodium bicarbonate 0.81 g/L, ammonium chloride 0.16 g/L, potassium chloride 0.03 g/L, and sodium chloride 0.45 g/L) with U(VI) 0.01 g/L	Anaerobic continuous column	50 days	7.5	50 mL sludge (inoculum) with 100 g woodchips and 100 g S(0)	Electron acceptors: V(V) and U(VI); electron donor: S(0) and woodchips	Sludge from an aquifer near V-tailings pond of V-mine in Panzhihua (China)	ICP-MS (for total V quantification) and spectrophotometric method (for V(V) quantification)	Liu et al. (2022)
	10 mg/L V(V)	4 mg/L V(V)/63.4% removed	Freshwater environment medium with U(VI) 0.05 g/L		38 days (51–88 days)						
	50 mg/L V(V)	24 mg/L V(V)/52.2% removed	Freshwater environment medium with U(VI) 0.01 g/L		21 days (89–109 days)						
	10 mg/L V(V)	3 mg/L V(V)/69.1% removed	Freshwater environment medium with U(VI) 0.01 g/L		31 days (110–140 days)						
Microbial community (e.g., *Thiobacillus, Desulfovibrio* and *Thermomonas*)	50 mg/L V(V)	18 mg/L V(V)/63.6% removed	Synthetic groundwater (sodium bicarbonate 0.50 g/L, calcium chloride 0.25 g/L, ammonium chloride 0.04 g/L, magnesium chloride 1.06 g/L, sodium chloride 0.45 g/L, potassium chloride 0.03 g/L, and monopotassium phosphate 0.03 g/L)	22 °C, anaerobic condition with solution periodically refreshed in operational cycles of 7 days	49 days	8.1	Aquifer sediments from V production region (China)	Electron acceptor: V(V); electron donor: pyrrhotite (sulfate)	Controls: (1) control with sterilized inoculum and (2) without inoculum	Spectrophotometric method (for V(V) quantification)	He et al. (2023)
		28 mg/L V(V)/44.1% removed			56 days (49–105 days)						
		38 mg/L V(V)/25% removed			42 days (105–147 days)						
		43 mg/L V(V)/13.2% removed			63 days (147–210 days)						
*Bacillus* sp. PFYN01 (intact cells)	50 mg/L V(V)	48 mg/L V(V)/4% removed	Nutrient Broth (NB) medium (beef extract 3 g, tryptone 10 g, and sodium chloride 5 g)	30 °C, 150 rpm	2 days (48 h)	8	Inoculum with 2% of precultured in NB medium for 12 h	Electron acceptor: V(V); electron donor: EPS	Strain isolated from Majiatian V tailing reservoir in Panzhihua (China)	Spectrophotometric method (for V(V) quantification)	Zhou et al. (2022)
	100 mg/L V(V)	90 mg/L V(V)/10% removed									
	150 mg/L V(V)	134 mg/L V(V)/11% removed									
	200 mg/L V(V)	184 mg/L V(V)/8% removed									
EPS—*Bacillus* sp. PFYN01 (EPS-free cells)	50 mg/L V(V)	48 mg/L V(V)/4% removed	NB medium	30 °C, 150 rpm	2 days (48 h)	8	Inoculum with 2% of precultured in NB medium for 12 h	Electron acceptor: V(V); electron donor: EPS	Strain isolated from Majiatian V tailing reservoir in Panzhihua (China)	Spectrophotometric method (for V(V) quantification)	Zhou et al. (2022)
	100 mg/L V(V)	95 mg/L V(V)/4% removed									
	150 mg/L V(V)	145 mg/L V(V)/3% removed									
	200 mg/L V(V)	195 mg/L V(V)/2.5% removed									
*Streptomyces microflavus*	25 mg/L V(V)	2 mg/L V(V)/92% removed	Synthetic groundwater with ethanol 0.3 g/L	30 °C, anaerobic condition	12 days	ND	OD_600_ = 0.5	Electron acceptor: V(V); electron donor: ethanol	Strain provided by Fujian Agriculture and Forestry University	Spectrophotometric method (for V(V) quantification)	Wang S. et al. (2023)
	50 mg/L V(V)	5 mg/L V(V)/90% removed									
	75 mg/L V(V)	17 mg/L V(V)/77% removed									
	100 mg/L V(V)	27 mg/L V(V)/73% removed									
*Bacillus megaterium* R01	300 mg/L V(V)	300 mg/L V(V)/0% removed	LB medium with 0.3 g/L sodium pyruvate	30 °C, 150 rpm	7 days	7	OD_600_ = 0.5	Electron acceptor: V(V); electron donor: carbon source	Strain isolated from a stone coal mine region in Yiyang City, Hunan Province (China)	Spectrophotometric method (for V(V) quantification)	Huang et al. (2025)
		285 mg/L V(V)/5% removed	LB medium with 0.3 g/L ethanol								
		276 mg/L V(V)/8% removed	LB medium with 0.3 g/L sorbitol								
		270 mg/L V(V)/10% removed	LB medium with 0.3 g/L sodium lactate								
		270 mg/L V(V)/10% removed	LB medium with 0.3 g/L mannitol								
		255 mg/L V(V)/15% removed	LB medium with 0.3 g/L glucose								
		246 mg/L V(V)/18% removed	LB medium with 0.3 g/L sodium citrate								
		237 mg/L V(V)/21% removed	LB medium with 0.3 g/L seignette salt								
		225 mg/L V(V)/25% removed	LB medium with 0.3 g/L fructose								
		135 mg/L V(V)/55% removed	LB medium with 0.3 g/L sucrose								
Microbial community (*Acinetobacter, Dechlorobacter, Denitratisoma* and *Nitrospira*)	5 mg/L V(V)	0.48 mg/L V/90% removed (0.38 mg/L V(V)/92% removed)	Wastewater blend (ammonium chloride 0.03 g/L, monopotassium phosphate 0.006 g/L, sodium bicarbonate 0.3 g/L, magnesium sulfate 0.1 g/L, calcium chloride 0.01 g/L, and trace element solution 1 mL/L)	24 °C, reactor system with continuous flow with 0.008 g/L dissolved oxygen	30 days (20–50 days)	7.5	Membrane-aerated biofilm	Electron acceptors: V(V) and nitrate; electron donor: chemical oxygen demand (COD) and EPS	During the experiment, concentrations of V(V), nitrate, and COD were determined	ICP-MS (for total V quantification) and spectrophotometric method (for V(V) quantification)	Sun et al. (2025)
	20 mg/L V(V)	1.79 mg/L V/91% removed (1.54 mg/L V(V)/92% removed)			30 days (50–80 days)						
		1.08 mg/L V/95% removed (0.94 mg/L V(V)/95% removed)			20 days (80–100 days)						
Microbial community (e.g., *Syntrophobacter*, *Spirochaeta*, *Geobacter*)	50 mg/L V(V)	35 mg/L V(V)/30.0% removed	Synthetic medium (sodium bicarbonate 0.50 g/L, ammonium chloride 0.05 g/L, calcium chloride 0.25 g/L, magnesium chloride 1.51 g/L, sodium chloride 0.45 g/L, potassium chloride 0.03 g/L, monopotassium phosphate 0.03 g/L) with S(0) 5 g	Anaerobic condition	6 days (144 h)	8	50 mL anaerobic sludge collected from a brewery anaerobic system without V(V) exposure (China)	Electron acceptor: V(V); electron donor: S(0)	Bioreactors with 200 mL of synthetic medium with three consecutive operating cycles and V(V) exposure	ICP-MS (for total V quantification) and XPS (for V valence state analysis)	Wang et al. (2021)
		29 mg/L V(V)/42.6% removed	Synthetic medium with acetate 0.06 g/L					Electron acceptor: V(V); electron donor: acetate			
		12 mg/L V(V)/76.9% removed	Synthetic medium with S(0) 5 g and acetate 0.06 g/L					Electron acceptor: V(V); electron donors: acetate and S(0)			
	25 mg/L V(V)	0 mg/L V(V)/100% removed	Synthetic medium with S(0) 5 g and acetate 0.06 g/L	Anaerobic condition	2 days (48 h)			Electron acceptor: V(V); electron donors: acetate and S(0)			
	50 mg/L V(V)	8 mg/L V(V)/83.9% removed									
	75 mg/L V(V)	25 mg/L V(V)/67.1% removed									
	100 mg/L V(V)	34 mg/L V(V)/66.2% removed									
	50 mg/L V(V)	2 mg/L V(V)/46.4% removed	Synthetic medium with S(0) 5 g and acetate 0.03 g/L								
		6 mg/L V(V)/88.3% removed	Synthetic medium with S(0) 5 g and acetate 0.09 g/L								
		30 mg/L V(V)/40% removed	Synthetic medium with S(0) 5 g and acetate 0.12 g/L								
Microbial community (e.g., *Geobacter, Petrimonas, Rhodococcus,* and *Sedimentibacter*)	10 mg/L V(V)	0.1 mg/L V(V)/98.8% removed	Synthetic medium (disodium phosphate 10.55 g/L, potassium phosphate 1.5 g/L, ammonium chloride 0.31 g/L, magnesium sulfate 0.1 g/L, potassium chloride 0.13 g/L, vitamin solution 10 mL/L, and trace mineral solution 5 mL/L) with acetate 1 g/L	Electric-stimulated reactor	49 days	ND	Anaerobic activated sludge	Electron acceptor: V(V); electron donor: acetate	Operated with an intermittent power supply (12 h-ON/12 h-OFF) at 0.2 V	ND	Jia et al. (2025)
	50 mg/L V(V)	7.5 mg/L V(V)/85% removed			56 days (49–105 days)						
	50 mg/L V(V)	2.8 mg/L V(V)/94.4% removed			45 days (105–150 days)						
Microbial community (e.g., *Petrimonas, Sedimentibacter, Geobacter, Petrimonas, Rhodococcus,* and *Sedimentibacter*)	10 mg/L V(V)	0.02 mg/L V(V)/99.8% removed	Synthetic medium with acetate 1 g/L	Electric-stimulated reactor	49 days	ND	Anaerobic activated sludge and anode effluent	Electron acceptor: V(V); electron donor: acetate	Operated with an intermittent power supply (12 h-ON/12 h-OFF) at 0.2 V	ND	Jia et al. (2025)
	50 mg/L V(V)	5 mg/L V(V)/90% removed			56 days (49–105 days)						
	50 mg/L V(V)	2.1 mg/L V(V)/95.8% removed			45 days (105–150 days)						
Microbial community (*Deltaproteobacteria, Anaerolineae, Campylobacteria, Bacteroidia* and *Gammaproteobacteria*)	10 mg/L V(V)	0.08 mg/L V(V)/99.2% removed	Synthetic groundwater solution (ammonium chloride 0.16 g/L, calcium chloride 0.25 g/L, magnesium chloride 1.06 g/L, sodium chloride 0.45 g/L, potassium chloride 0.03 g/L, sodium bicarbonate 0.36 g/L, and monopotassium phosphate 0.03 g/L) with Cr(VI) 0.01 g/L	22 °C, anaerobic column	165 days	ND	80 mL anaerobic microbial consortium	Electron acceptors: V(V) and Cr(VI); electron donor: S(0)	Inoculum mixed with 100 g of S(0) and quartz sand; anaerobic consortium from brewery wastewater treatment (China)	Spectrophotometric method (for V(V) quantification)	Shi et al. (2020)
	50 mg/L V(V)	2.5 mg/L V(V)/95% removed	Synthetic groundwater solution with Cr(VI) 0.01 g/L		44 days (166–209 days)						
	10 mg/L V(V)	1 mg/L V(V)/90% removed	Synthetic groundwater solution with Cr(VI) 0.05 g/L		32 days (210–241 days)						
	10 mg/L V(V)	1.17 mg/L V(V)/88.3% removed	Synthetic groundwater solution with Cr(VI) 0.01 g/L		35 days (242–276 days)						
Deep-sea microbial community (e.g., *Burkholderia, Caballeronia*, and *Paraburkholderia*)	100 mg/L V(V)	2 mg/L V(V)/98.02% removed	Deep-sea medium (glucose 0.75 g/L, monosodium phosphate 4.97 g/L, disodium phosphate 2.75 g/L, ammonium chloride 0.31 g/L, potassium chloride 0.13 g/L, vitamin solution 1.25 mL/L, trace mineral element solution 12.5 mL/L and salinity 30%)	30 °C, 150 rpm	10 days	7.5	1% (V/V) deep-sea hydrothermal vent sediments	Electron acceptor: V(V); electron donor: glucose	Control: high-temperature sterilized deep-sea hydrothermal vent sediments	Spectrophotometric method (for V(V) quantification)	Ma et al. (2024)
	100 mg/L V(V)	5.9 mg/L V(V)/94.14% removed	Deep-sea medium with cadmium chloride 0.02 g/L								
Microbial community (e.g., *Geothrix* and *Rudaea*)	2 mg/L V(V)	1.8 mg/L V(V)/8.4% removed	Mineral salt medium (calcium chloride 0.001 g/L, sodium bicarbonate 0.3 g/L, magnesium sulfate 0.005 g/L, potassium dihydrogen phosphate 0.2 g/L, disodium phosphate 0.4 g/L, acid trace element solution 1 mL/L, and alkaline trace element solution 1 mL/L)	Anaerobic condition	22 days	7.0	10 mL activated sludge internally circulated for 48 h	Electron acceptor: V(V); electron donor: ethane	Membrane biofilm reactor (MBfR) that delivered ethane with a pressure of 6 psig directly to a biofilm	ICP-MS (for total V quantification) and spectrophotometric method (for V(V) quantification)	Chi et al. (2024)
	2 mg/L V(V)	1.6 mg/L V(V)/18.8% removed		Aerobic condition (influent O_2_ 100 mg/L)	23 days (22–44 days)						
	2 mg/L V(V)	0.3 mg/L V(V)/87.5% removed			37 days (45–81 days)						
	2 mg/L V(V)	0.2 mg/L V(V)/89.2% removed			67 days (81–147 days)						
	2 mg/L V(V)	0.2 mg/L V(V)/89.6% removed			21 days (148–168 days)						
	4 mg/L V(V)	0.3 mg/L V(V)/92.3% removed			18 days (169–186 days)						
	8 mg/L V(V)	0.5 mg/L V(V)/93.5% removed			45 days (187–231 days)						
Sediments microbiome (e.g., *Sulfuricurvum* sp., *Pseudomonas cremoris*, *Thiomonas* sp., *Achromobacter* sp., *Methanobacterium* sp., and *Anaeromyxobacter* sp.)	10 mg/L V(V)	0.2 mg/L V(V)/98% removed	Synthetic wastewater (monopotassium phosphate 0.03 g/L, potassium chloride 0.03 g/L, sodium chloride 0.45 g/L, magnesium chloride 1.06 g/L, sodium bicarbonate 0.81 g/L, and calcium chloride 0.25 g/L)	Column with a rate of 0.13 mL/min for 226 days	93 days	ND	50 mL of aquifer sediments from a V-tailing reservoir (China) (used as inoculum)	Electron acceptor: V(V); electron donor: S(0)	Inoculum mixed with 200 g of S(0) and granular graphite	Spectrophotometric method (for V(V) quantification)	Yan et al. (2023)
	50 mg/L V(V)	1.7 mg/L V(V)/97% removed			36 days (94–129 days)						
	100 mg/L V(V)	37 mg/L V(V)/63% removed			58 days (130–187 days)						
	50 mg/L V(V)	19 mg/L V(V)/63% removed			39 days (188–226 days)						
Microbial community (e.g., *Pseudomonas*, *Shinella*, *Azospira*, *Acidovorax, Acinetobacter* and *Exiguobacterium*)	10 mg/L V(V)	0.3 mg/L V(V)/96.8% removed	V-bearing smelting wastewater with ammonium 0.01 g/L and COD 0.1 g/L	Membrane biofilm reactor (MBfR)	24 days (1–24 days)	8	3 mL culture acquired from a parent anaerobic V(V) reduction bioreactor with acetate	Electron acceptors: V(V) and nitrate; electron donor: acetate	Controls (sealed serum bottles): (1) inoculum with V(V) 10 mg/L and ammonium 10 mg/L (anoxic conditions), (2) inoculum with V(V) 10 mg/L, ammonium 10 mg/L, and acetate (anoxic conditions), (3) inoculum with V(V) 10 mg/L, ammonium 10 mg/L and dissolved oxygen (aerobic conditions), (4) inoculum of *Pseudomonas stutzeri* pure culture with V(V) 10 mg/L and ammonium 10 mg/L	ICP-MS (for total V quantification) and spectrophotometric method (for V(V) quantification)	Li et al. (2022)
	10 mg/L V(V)	1 mg/L V(V)/90.3% removed	V-bearing smelting wastewater with ammonium 0.01 g/L and COD 0.1 g/L		30 days (25–54 days)						
	50 mg/L V(V)	37 mg/L V(V)/26.5% removed	V-bearing smelting wastewater with ammonium 0.01 g/L and COD 0.1 g/L		59 days (55–113 days)						
	10 mg/L V(V)	1 mg/L V(V)/90.1% removed	V-bearing smelting wastewater with ammonium 0.05 g/L and COD 0.1 g/L		38 days (114–151 days)						
	10 mg/L V(V)	4 mg/L V(V)/60.5% removed	V-bearing smelting wastewater with ammonium 0.01 g/L and COD 0.05 g/L		29 days (152–180 days)						
Microbial community (e.g., *Bacteroidetes*, *Paludibacter*, and *Thauera*)	75 mg/L V(V)	1 mg/L V(V)/98.7% removed	Nutrient solution (ammonium chloride 0.16 g/L, calcium chloride 0.25 g/L, magnesium chloride 1.06 g/L, sodium chloride 0.45 g/L, potassium chloride 0.03 g/L, sodium bicarbonate 0.81 g/L, potassium phosphate 0.03 g/L) with glucose	25 °C, anaerobic condition	10 days	ND	20 mL sludge domesticated for 30 days	Electron acceptor: V(V); electron donor: glucose	Sludge from Qinghe Wastewater Treatment Plant, Beijing (China)	Spectrophotometric method (for V(V) quantification)	Hao et al. (2021a)
		7 mg/L V(V)/90.3% removed	Nutrient solution with sawdust 0.075 g/L					Electron acceptor: V(V); electron donor: sawdust			
		35 mg/L V(V)/53.2% removed	Groundwater					Electron acceptor: V(V); electron donor: groundwater	Sludge from Qinghe Wastewater Treatment Plant, Beijing (China) and groundwater from China University of Geosciences, Beijing (China)		
		13 mg/L V(V)/82.6% removed	Groundwater with sterilized phosphate rock 0.2 g/L and sterilized medical stone 0.2 g/L					electron acceptor: V(V); electron donor: groundwater			
Microbial community (e.g., *Pedobacter, Enterobacter,* and *Bacillus*)	11 mg/L V(V)	3 mg/L V(V)/73% removed	Synthetic wastewater with corncobs 0.05 g/L	30 °C, anaerobic conditions	10.4 days (250 h)	ND	Anaerobic enrichment culture of agro-industrial with nitrate 0.1 g/L and V(V) 0.01 g/L	Electron acceptors: V(V) and nitrate	Sludge from pre-anoxic zone of the Qinghe Wastewater Treatment Plant, Beijing (China)	Spectrophotometric method (for V(V) quantification)	Wang H. et al. (2023)
	10 mg/L V(V)	2 mg/L V(V)/80% removed			5.8 days (140 h) (251–390 h)						
	10 mg/L V(V)	3 mg/L V(V)/70% removed			6.3 days (150 h) (391–540 h)						
Microbial community (e.g., *Pedobacter, Enterobacter,* and *Bacillus*)	11 mg/L V(V)	2 mg/L V(V)/82% removed	Synthetic wastewater with corn straw 0.05 g/L	30 °C, anaerobic conditions	10.4 days (250 h)	ND	Anaerobic enrichment culture of agro-industrial with nitrate 0.1 g/L and V(V) 0.01 g/L	Electron acceptors: V(V) and nitrate	Sludge from pre-anoxic zone of the Qinghe Wastewater Treatment Plant, Beijing (China)	Spectrophotometric method (for V(V) quantification)	Wang H. et al. (2023)
	10 mg/L V(V)	9 mg/L V(V)/10% removed			5.8 days (140 h) (251–390 h)						
	10 mg/L V(V)	5 mg/L V(V)/50% removed			6.3 days (150 h) (391–540 h)						
Microbial community (e.g., *Pedobacter, Enterobacter,* and *Bacillus*)	11 mg/L V(V)	10 mg/L V(V)/9% removed	Synthetic wastewater with woodchips 0.05 g/L	30 °C, anaerobic conditions	10.4 days (250 h)	ND	Anaerobic enrichment culture of agro-industrial with nitrate 0.1 g/L and V(V) 0.01 g/L	Electron acceptors: V(V) and nitrate	Sludge from pre-anoxic zone of the Qinghe Wastewater Treatment Plant, Beijing (China)	Spectrophotometric method (for V(V) quantification)	Wang H. et al. (2023)
	10 mg/L V(V)	9 mg/L V(V)/10% removed			5.8 days (140 h) (251–390 h)						
	10 mg/L V(V)	12 mg/L V(V)/0% removed			6.3 days (150 h) (391–540 h)						
Microbial community (e.g., *Pedobacter, Enterobacter,* and *Bacillus*)	11 mg/L V(V)	2 mg/L V(V)/82% removed	Synthetic wastewater with wheat straw 0.05 g/L	30 °C, anaerobic conditions	10.4 days (250 h)	ND	Anaerobic enrichment culture of agro-industrial with nitrate 0.1 g/L and V(V) 0.01 g/L	Electron acceptors: V(V) and nitrate	Sludge from pre-anoxic zone of the Qinghe Wastewater Treatment Plant, Beijing (China)	Spectrophotometric method (for V(V) quantification)	Wang H. et al. (2023)
	10 mg/L V(V)	2 mg/L V(V)/80% removed			5.8 days (140 h) (251–390 h)						
	10 mg/L V(V)	3 mg/L V(V)/70% removed			6.3 days (150 h) (391–540 h)						
Microbial community (e.g., *Sulfurospirllum, Geobacter* and *Wolinella*)	50 mg/L V(V)	2 mg/L V(V)/96% removed	Growth medium (potassium chloride 0.1 g/L, ammonium chloride 1.5 g/L, potassium phosphate 0.6 g/L, calcium chloride 0.1 g/L, magnesium chloride 0.1 g/L, trace elements solution 10 mL/L, vitamin solution 5 mL/L, fumaric acid 4.64 g/L, and carboxymethyl cellulose 3.8 g/L)	30 °C, two-chamber microbial fuel cells: anode chamber with the medium, inoculum and V(V) solution; cathode chamber with 50 mM potassium ferricyanide in 100 mM phosphate buffer	1.2 days (28 h)	5.7	Inoculum size 21.4% (inoculum: 5 mL of activated sludge in 25 mL of growth medium)	Electron acceptors: V(V) and fumaric acid; electron donor: carboxymethyl cellulose	Activated sludge obtained from sewage treatment plant in Zhaoqing, Guangzhou (China)	Spectrophotometric method (for V(V) quantification)	Fan et al. (2025)
	100 mg/L V(V)	5 mg/L V(V)/95% removed									
	150 mg/L V(V)	11 mg/L V(V)/93% removed									
	200 mg/L V(V)	14 mg/L V(V)/93% removed									
	250 mg/L V(V)	43 mg/L V(V)/83% removed									
	300 mg/L V(V)	144 mg/L V(V)/52% removed									
	400 mg/L V(V)	332 mg/L V(V)/17% removed									
	500 mg/L V(V)	440 mg/L V(V)/12% removed									
Microbial community (e.g., *Geobacter, Sphingomonas, Bacillus, and Anaerolinea*)	10 mg/L V(V)	0.1 mg/L V/99% removed	Synthetic groundwater (sodium bicarbonate 0.81 g/L, calcium chloride 0.25 g/L, sodium acetate 1.03 g/L, ammonium chloride 0.16 g/L, potassium chloride 0.03 g/L, potassium phosphate 0.03 g/L, and magnesium chloride 1.06 g/L).	Anaerobic condition	30 days	ND	Inoculum (150 g of soil sample)	Electron acceptor: V(V); electron donor: acetate	Soil sample from V-smelter of Huaihua Fengmutan (China)	ICP-OES (for total V quantification)	Zhang H. et al. (2021)
	50 mg/L V(V)	3 mg/L V/94.6% removed			25 days (31–55 days)						
	10 mg/L V(V)	4 mg/L V/61.9% removed			30 days (56–85 days)						
	10 mg/L V(V)	1 mg/L V/88.8% removed	Synthetic groundwater with nitrate 0.045 g/L		25 days (86–110 days)			Electron acceptors: V(V) and nitrate; electron donor: acetate			
	10 mg/L V(V)	0 mg/L V/100% removed	Synthetic groundwater		30 days (111–140 days)			Electron acceptor: V(V); electron donor: acetate			
Microbial community (e.g., *Methylomonas, Stenotrophomonas* and *Steroidobacter*)	61 mg/L V(V)	2.5 mg/L V/96% removed	Synthetic groundwater (ammonium chloride 0.16 g/L, calcium chloride 0.25 g/L, magnesium chloride 1.06 g/L, sodium chloride 0.45 g/L, potassium chloride 0.03 g/L, potassium phosphate 0.03 g/L)	22 °C, anaerobic condition, in the dark with injection of methane 0.08 g/	7 days	ND	20 mL anaerobic sludge from wastewater treatment	Electron acceptor: V(V); electron donor: methane	Sludge from wastewater treatment Yanjing Brewery, in Beijing (China) Control: abiotic control reactor with V(V)	ICP-MS (for total V quantification)	Zhang et al. (2020)
		1.2 mg/L V/98% removed			7 days (7–14 days)						
		2.5 mg/L V/96% removed			7 days (14–21 days)						
		1.2 mg/L V/98% removed		22 °C, anaerobic condition, in the dark	7 days (21–28 days)			Electron acceptor: V(V)			
		36.5 mg/L V/40% removed		22 °C, anaerobic condition, in the dark with injection of methane 0.08 g/and nitrate 0.03 g/L	7 days (28–35 days)			Electron acceptors: V(V) and nitrate; electron donor: methane			
		39 mg/L V/36% removed			7 days (35–42 days)						
		32 mg/L V/48% removed			7 days (42–49 days)						
Microbial community (e.g., *Methylomonas, Stenotrophomonas* and *Steroidobacter*)	122 mg/L V(V)	15 mg/L V/88% removed	Synthetic groundwater	22 °C, anaerobic condition, in the dark with injection of methane 0.08 g/	7 days	ND	20 mL anaerobic sludge from wastewater treatment	Electron acceptor: V(V); electron donor: methane	Sludge from wastewater treatment Yanjing Brewery, in Beijing (China) Control: abiotic control reactor with V(V)	ICP-MS (for total V quantification)	Zhang et al. (2020)
		12 mg/L V/90% removed			7 days (7–14 days)						
		13 mg/L V/89% removed			7 days (14–21 days)						
		15 mg/L V/88% removed		22 °C, anaerobic condition, in the dark	7 days (21–28 days)			Electron acceptor: V(V)			
		88 mg/L V/28% removed		22 °C, anaerobic condition, in the dark with injection of methane 0.08 g/and nitrate 0.06 g/L	7 days (28–35 days)			Electron acceptors: V(V) and nitrate; electron donor: methane			
		92 mg/L V/25% removed			7 days (35–42 days)						
		83 mg/L V/32% removed			7 days (42–49 days)						
Microbial community (e.g., *Methylomonas, Stenotrophomonas* and *Steroidobacter*)	183 mg/L V(V)	37 mg/L V/80% removed	Synthetic groundwater	22 °C, anaerobic condition, in the dark with injection of methane 0.08 g/	7 days	ND	20 mL anaerobic sludge from wastewater treatment	Electron acceptor: V(V); electron donor: methane	Sludge from wastewater treatment Yanjing Brewery, in Beijing (China) Control: abiotic control reactor with V(V)	ICP-MS (for total V quantification)	Zhang et al. (2020)
		33 mg/L V/82% removed			7 days (7–14 days)						
		36 mg/L V/80% removed			7 days (14–21 days)						
		33 mg/L V/82% removed		22 °C, anaerobic condition, in the dark	7 days (21–28 days)			Electron acceptor: V(V)			
		161 mg/L V/12% removed		22 °C, anaerobic condition, in the dark with injection of methane 0.08 g/and nitrate 0.09 g/L	7 days (28–35 days)			Electron acceptors: V(V) and nitrate; electron donor: methane			
		159 mg/L V/13% removed			7 days (35–42 days)						
		157 mg/L V/14% removed			7 days (42–49 days)						
Microbial community (e.g., *Firmicutes, Bacteroidetes, Chloroflexi, Tenericutes, Proteobacteria, Actinobacteria, Acidobacteria* and *Spirochaetae*)	51.4 mg/L V(V)	9 mg/L V(V)/82.7% removed	Nutrient solution (calcium chloride 0.25 g/L, potassium chloride 0.03 g/L, potassium phosphate 0.03 g/L, magnesium chloride 1.06 g/L, sodium chloride 0.45 g/L, sodium bicarbonate 0.81 g/L, ammonium chloride 0.16 g/L, glucose 0.75 g/L, and sodium metavanadate 0.18 g/L)	22 °C, reactor with 100 g of soil and 200 mL nutrient solution	2.5 days (60 h)	7.3	Domestication of soil microbiome for 240 days, reactors refreshed by nutrient solution every 3 days	Electron acceptors: V(V) and nitrate; electron donor: S(0) (from soil)	Farmland soil sample near the V ore mining area in Sichuan province (China)	ICP-MS (for total V quantification) and spectrophotometric method (for V(V) quantification)	Hao et al. (2021b)
Microbial community (e.g., *Firmicutes, Bacteroidetes, Chloroflexi, Tenericutes, Proteobacteria, Actinobacteria, Acidobacteria* and *Spirochaetae*)	272.0 mg/L V(V)	24 mg/L V(V)/91.3% removed	Nutrient solution	22 °C, reactor with 100 g of soil and 200 mL nutrient solution	2.5 days (60 h)	5.9	Domestication of soil microbiome for 240 days, reactors refreshed by nutrient solution every 3 days	Electron acceptors: V(V) and nitrate; electron donor: S(0) (from soil)	Farmland soil sample near the V ore mining area in Hunan province (China)	ICP-MS (for total V quantification) and spectrophotometric method (for V(V) quantification)	Hao et al. (2021b)
Microbial community (e.g., *Firmicutes, Bacteroidetes, Chloroflexi, Tenericutes, Proteobacteria, Actinobacteria, Acidobacteria* and *Spirochaetae*)	57.4 mg/L V(V)	4 mg/L V(V)/9.4% removed	Nutrient solution	22 °C, reactor with 100 g of soil and 200 mL nutrient solution	2.5 days (60 h)	8.2	Domestication of soil microbiome for 240 days, reactors refreshed by nutrient solution every 3 days	Electron acceptors: V(V) and nitrate; electron donor: S(0) (from soil)	Farmland soil sample near the V ore mining area in Hubei province (China)	ICP-MS (for total V quantification) and spectrophotometric method (for V(V) quantification)	Hao et al. (2021b)
Microbial community (e.g., *Firmicutes, Bacteroidetes, Chloroflexi, Tenericutes, Proteobacteria, Actinobacteria, Acidobacteria* and *Spirochaetae*)	41.6 mg/L V(V)	1 mg/L V(V)/98.4% removed	Nutrient solution	22 °C, reactor with 100 g of soil and 200 mL nutrient solution	2.5 days (60 h)	7.9	Domestication of soil microbiome for 240 days, reactors refreshed by nutrient solution every 3 days	Electron acceptors: V(V) and nitrate; electron donor: S(0) (from soil)	Farmland soil sample near V ore mining area in Guizhou province (China)	ICP-MS (for total V quantification) and spectrophotometric method (for V(V) quantification)	Hao et al. (2021b)
*Enterococcus faecalis*	100 mg/L V(V)	50 mg/L V(V)/50% removed	Groundwater solution (calcium trichloride 0.25 g/L, magnesium chloride 1.06 g/L, sodium chloride 0.45 g/L, potassium chloride 0.03 g/L, sodium bicarbonate 0.81 g/L, ammonium chloride 0.16 g/L, monopotassium phosphate 0.03 g/L) with sodium pyruvate 0.3 g/L	30 °C, anaerobic conditions and protection from light	7 days	7.0	OD_600_ = 1.35	Electron acceptor: V(V); electron donor: carbon sources	During the experiment, different conditions were tested: carbon source and pH	Spectrophotometric method (for V(V) quantification) and XPS (for V valence state analysis)	Zhang L. et al. (2025)
		30 mg/L V(V)/70% removed	Groundwater solution with glucose 0.3 g/L								
		14.6 mg/L V(V)/85% removed	Groundwater solution with sucrose 0.3 g/L								
	250 mg/L V(V)	218 mg/L V(V)/13% removed	Groundwater solution with total organic carbon 0.3 g/L			4.0					
		19 mg/L V(V)/92% removed				5.0					
		12 mg/L V(V)/95% removed				6.0					
		25 mg/L V(V)/90% removed				7.0					
		30 mg/L V(V)/88% removed				8.0					
		31 mg/L V(V)/88% removed				9.0					
		56 mg/L V(V)/78% removed				10.0					
Nanoparticles biogenesis											
*Shewanella* sp. strain HN-41	122 mg/L V(V)	78 mg/L V(V)/36% V(V) removal	HEPES-buffered basal medium with sodium lactate 1.12 g/L	30 °C, anaerobic conditions	5 days (120 h)	7	OD_600_ = 0.1	Electron acceptor: V(V); electron donor: lactate	Strain incubated in LB medium (tryptone 10 g/L, yeast extract 5 g/L, sodium chloride 10 g/L) at 30 °C, 180 rpm for 18 h	ICP-OES (for total V quantification)	Ko et al. (2025)
		Biogenic VO_2_ nanoparticles								TEM, SEM-EDS, XRD and DSC	

AAS, atomic absorption spectrometry; DSC, differential scanning calorimetry; IC, ion chromatography; ICP-AES, inductively coupled plasma atomic emission spectrometry; ICP-MS, inductively coupled plasma mass spectrometry; ICP-OES, inductively coupled plasma optical emission spectroscopy; SEM-EDS, scanning electron microscopy/energy dispersive spectroscopy; TEM, transmission electron microscope; XPS, X-ray photoelectron spectroscopy; XRD, X-ray diffraction; 3DEEM, three-dimensional excitation emission matrix fluorescence spectroscopy; ND, no data.

The V-bioaccumulation studies had an extra challenge in comparing results between different articles due to the use of varying units for bioaccumulation. One article reports results in μg V/mg total protein, while another uses μg V/g dry weight of cells (Almeida et al., 2020; Yuliani et al., 2024). However, neither article provides information regarding protein concentration or mass of dry cells for each sample, which limits the ability to compare results across the literature (Almeida et al., 2020; Yuliani et al., 2024). The study focused on the V(V)-bioaccumulation described an *Ochrobactrum tritici* strain able to accumulate intracellularly 3.5 μg V/mg total protein when exposed for 3 h (in the middle of the exponential growth phase) to a concentration of 15 mM V(V) (Almeida et al., 2020). Another study tested the V-bioaccumulation using two strains at different pH values of 3, 5, and 7, and with two oxidation states of V: V(V) and V(IV) (Yuliani et al., 2024). The strain *Vibrio* sp. CD2-102 outperformed *Pseudoalteromonas* sp. CD2-88, with both strains accumulating over 4,000 μg V/g dry weight of cells when exposed to 0.5 mM V(V) and over 260 μg V/g dry weight of cells with 0.5 mM V(IV). For both strains, the highest bioaccumulation values were observed at pH 3 for V(V) and at pH 5 for V(IV) (Yuliani et al., 2024). Additionally, an experiment investigated the optimal pH for V-bioleaching by using a mutagenized *Bacillus mucilaginosus* strain along with a shale sample containing 0.75% V (Tian et al., 2023). The pH values tested ranged from 6.0 to 9.0, with the best bioleaching efficiency of 68.5% achieved at pH 7.5 (Tian et al., 2023). Regarding V(V)-bioreduction, Zhang B. et al. (2021) explored various pH values from 5.0 to 8.0 using a *Lactococcus raffinolactis* strain, which successfully bioreduced 88% of the V(V) present in solution at a neutral pH. The highest efficiency for V-removal was observed at pH 7 with the *Acidovorax* sp. strain BoFeN1 (Fei et al., 2024). However, an *Enterococcus faecalis* strain was more effective at removing V(V) at a lower pH of 6.0 (Zhang H. et al., 2025).

The pH was not the only parameter evaluated to determine the optimal conditions for V-biomobilization. For example, various carbon sources were tested for their impact on V-removal. A study involving the *Bacillus megaterium* R01 strain compared 10 different carbon sources at the same concentration (0.3 g/L). The carbon sources included sodium pyruvate, ethanol, sorbitol, sodium lactate, mannitol, glucose, sodium citrate, seignette salt, fructose, and sucrose. Among these, sucrose demonstrated the highest V-removal efficiency, achieving a value of 55% (Huang et al., 2025). Another study backed this finding, indicating that sucrose was the most effective carbon source for the *Enterococcus faecalis* strain, with an efficiency of 85%. In comparison, glucose and sodium pyruvate showed efficiencies of 70 and 50%, respectively (Zhang L. et al., 2025). A study by Wang H. et al. (2023) compared different carbon sources at a concentration of 0.05 g/L, including corn straw, corncobs, woodchips, and wheat straw. Their results showed that the corn straw-based carbon source yielded an efficiency of 82% (Wang H. et al., 2023).

Another parameter assessed in the selected literature was the addition of nitrate. Despite the varying nitrate concentrations and conditions tested, the results consistently showed that the lowest nitrate concentration tested yielded the highest V-biomobilization efficiencies (Zhang et al., 2020; He et al., 2021; Zhang H. et al., 2021; Tian et al., 2023). In the context of V-bioleaching, a mutagenized *Bacillus mucilaginosus* strain was studied alongside a range of nitrate concentrations from 0.5 to 4 g/L. The optimal nitrate concentration achieving maximum efficiency was found to be 0.5 g/L, resulting in an efficiency of 69% (Tian et al., 2023). In the context of V(V)-bioreduction, the highest observed efficiency occurred with nitrate supplementation at 0.05 g/L (He et al., 2021). In a recent study, Zhang H. et al. (2021) revealed that adding nitrate (0.045 g/L) led to a decrease in V-removal efficiency. An experiment investigated the capacity of an *Acidovorax* sp. strain to remove V(V). The strain achieved 76.5% removal in 120 h when exposed to 10 mg/L V(V) along with 10 mg/L nitrate, and nearly complete removal of V when exposed to only V(V) without nitrate (Fei et al., 2024). Another investigation indicated that incorporating nitrate reduced the V-removal rate, with concentrations varying from 0.03 g/L to 0.09 g/L (Zhang et al., 2020).

The effects of various heavy metals on V-removal were investigated. One study found that as the concentration of uranium (U(VI)) increased, the efficiency of V-removal decreased. The highest V-removal efficiency of 99.5% was obtained at the lowest concentration of 0.01 g/L U(VI). However, when the concentration was increased to 0.05 g/L U(VI), the efficiency dropped to 63.4% (Liu et al., 2022). Similarly, the influence of Cr(VI) was tested on V-removal in an anaerobic column (Shi et al., 2020). The results demonstrated a comparable pattern in V-removal, with the highest efficiency reaching 99.2% with a concentration of 0.01 g/L Cr(VI) (Shi et al., 2020). In another study, the impact of Cr(VI) on V-bioaccumulation was explored, revealing a different pattern (Almeida et al., 2020). The addition of 0.02 g/L Cr(VI) in the presence of a higher V(V) concentration (15 mM) increased V-bioaccumulation by 1.4 and 1.2 times for the bacterial strains *Ochrobactrum tritici* 5bvl1 and SCII24^T^, respectively. In contrast, when the V(V) concentration was lower (3 mM), the addition of Cr(VI) had little to no effect on V-bioaccumulation, with the V-concentration inside the cells remaining approximately identical (Almeida et al., 2020).

Some articles tested the impact of different initial concentrations of V(V) to evaluate the ability of bacteria to promote V-biomobilization, specifically focusing on bioreduction and removal. Studies investigating the removal of V(V) from liquid solutions using the isolated strains *Acidovorax* sp. BoFeN1, *Pseudogulbenkiania* sp. 2002, and *Streptomyces microflavus* showed higher V(V)-removal efficiencies at lower concentrations. The removal efficiencies were 89% (for 10 mg/L), 96% (for 10 mg/L), and 92% (for 25 mg/L), respectively (Fei et al., 2023; Wang S. et al., 2023). This trend was also observed in studies involving microbial communities, which achieved removal efficiencies of 96 and 100% at the lower concentrations of 50 mg/L and 25 mg/L, respectively (Wang et al., 2021; Fan et al., 2025). Additionally, a study with *Shewanella* sp. FDA-1 indicated that the lowest V(V)-bioreduction efficiency (35%) occurred at the highest V(V) concentration of 610 mg/L V(V) (Cheng et al., 2025). Thus, whether using isolated strains or exploring microbial communities, it appears that higher concentrations of V(V) lead to lower process efficiencies. In a separate 10-day experiment involving *Lactococcus raffinolactis,* different V(V) concentrations (25 mg/L, 50 mg/L, 75 mg/L, and 100 mg/L) were tested. This experiment yielded various V(V) removal efficiencies, with the highest being 88% with 50 mg/L V(V) (Zhang B. et al., 2021).

A study isolated a *Tepidibacter mesophilus* strain VROV1 from deep-sea sediments and explored the capacity to bioreduce V(V) (Kim et al., 2025). The authors describe that the VROV1 strain had a specific V(V) reduction rate of 2.8 pmol/cell/day, approximately 10 times higher than the Gram-positive V(V) reducer *Lactococcus raffinolactis* (0.16 pmol/cell/day) (Kim et al., 2025). A bioleaching process was promoted by a microbial consortium (*Acidovorax, Delftia* and *Pseudomonas* strains) to obtain a V leaching concentration of 0.680 mg/L, which corresponded to a V-concentration 7 times higher than the control condition (Zhang et al., 2022). A different consortium (mainly composed by *Acinetobacter, Dechlorobacter, Denitratisoma* and *Nitrospira* strains) was used in a membrane-aerated biofilm reactor system with continuous flow to promote the V(V)-bioreduction, achieving a final V(V) concentration of 0.9 mg/L (Sun et al., 2025).

A work explored *Acidithiobacillus thiooxidans* from a culture collection to bioleached V from a spent desulfurization catalyst sample (Mikoda et al., 2020). The authors reported 98.5% of V-bioleaching, using elemental sulfur (S(0)) as an electron donor. Other studies used this same electron donor and achieved even better V-biomobilization results. A microbial consortium from a brewery wastewater treatment was able to bioreduce 100% of V(V) present in a synthetic V(V)-contaminated groundwater (He et al., 2021). Other authors explored the microbial community from an anaerobic sludge to remove 99.5% of V(V) present in a freshwater environment medium (Liu et al., 2022). Acetate was also an electron donor used to promote V-biomobilization with different microbial communities, achieving some interesting results with V(V)-removal percentages higher than 90% (Zhang H. et al., 2021; Li et al., 2022; Jia et al., 2025).

Recent research over the past 5 years has enhanced the understanding of how bacteria interact with V, particularly in relation to redox transformations, intracellular accumulation, and the role of electron donors in V(V)-bioreduction. Earlier studies primarily focused on the ability of individual bacterial strains to interact with V under controlled laboratory conditions. However, more recent investigations have begun to examine cell-specific reduction rates, metabolic pathways associated with V transformation, and the effects of environmental factors such as nitrate availability and anaerobic conditions. Moreover, studies involving microbial consortia have shed light on synergistic interactions and community-level responses to V-exposure. Despite these advancements, considerable knowledge gaps still exist regarding the molecular mechanisms of V transport, the identity and regulation of enzymatic reduction systems, and how cells respond to V-induced stress. Additionally, inconsistencies in V speciation analysis continue to hinder our mechanistic understanding. To make further progress, it is crucial to harmonize methodologies, conduct evaluations in environmentally relevant and scalable conditions, and pursue integrative research that combines physiological, biochemical, and molecular approaches to fully elucidate the microbial processes driving V transformation.

## 6 Vanadium and resistance to antibiotics

Despite the relative lack of research on this topic in the literature, some studies have explored potential correlations between the presence of antibiotic resistance genes (ARG) and bacterial V-resistance. For instance, a study of samples contaminated by heavy metals, from lakes and wetlands in Iran, observed positive correlations between metal concentrations (including V) and specific ARGs, including tetracycline resistance genes (specifically *tetC* gene) and beta-lactamases (particularly SHV and OXA) (Komijani et al., 2021). However, as this study was based on correlation analyses, it does not establish a direct causal relationship between V-exposure and ARG enrichment, and other co-occurring metals or environmental stress agents may have contributed to the observed patterns. In another study, over 60% of multidrug-resistant bacteria isolated from an open dumpsite soil (including strains of *Bacillus subtilis*, *Bacillus cereus*, *Citrobacter freundii*, *Pseudomonas aeruginosa*, *Enterobacter* sp. and *Escherichia coli*) were found to be resistant to moderate V-concentrations (150 mg/L V) (Edet et al., 2023). However, the coexistence of antibiotic and V-resistance does not necessarily indicate shared or linked resistance mechanisms. In another study, the expression of polymyxin-resistance genes (*pmrE* and *pmrF*) in mutated *Escherichia coli* strains was linked with increased V(V)-resistance, suggesting that resistance mechanisms to certain antibiotics may overlap with those conferring V-tolerance (Joo et al., 2023). Nevertheless, it remains unclear whether this overlapping mechanism represents true cross-resistance, co-regulation, or indirect stress adaptation.

Although current evidence remains limited, it is possible that the existence of associations between V-exposure and antibiotic resistance traits. Future research should investigate whether V-resistance determinants are co-localized with ARGs on mobile genetic elements, potentially facilitating horizontal gene transfer under metal stress conditions. Moreover, long-term exposure assays and metagenomic analyses are needed to determine whether environmentally relevant V concentrations can drive the enrichment and dissemination of ARGs at the microbial community level. Understanding these interactions will be important for assessing the main ecological and health implications of V contamination.

## 7 Vanadium nanoparticles and other complexes

One of the articles selected for this review described the capacity to use bacteria for the biogenesis of V-nanoparticles (Ko et al., 2025). *Shewanella* sp. strain HN-41 synthesized V(IV)-nanoparticles (VO_2_ nanoparticles) with exposure to V(V) and lactate. The bacterial strain consumed lactate and promoted the bioreduction of V(V) to V(IV). The V(IV)-nanoparticles were produced on the cell surface and then secreted through membrane vesicles. This process was visualized with microscopy techniques. The V(IV)-nanoparticles obtained had a range of sizes between 1.5 and 9.5 nm and an average size of 4.3 nm.

While this review does not primarily focus on medical uses, it is important to note that there have been over 2,000 published articles in the last decade exploring V-applications in medical treatments. V-complexes and V-nanoparticles can potentially be applied for different treatments (Hashmi et al., 2023).

## 8 Knowledge gaps and future perspectives

**Figure 2:**
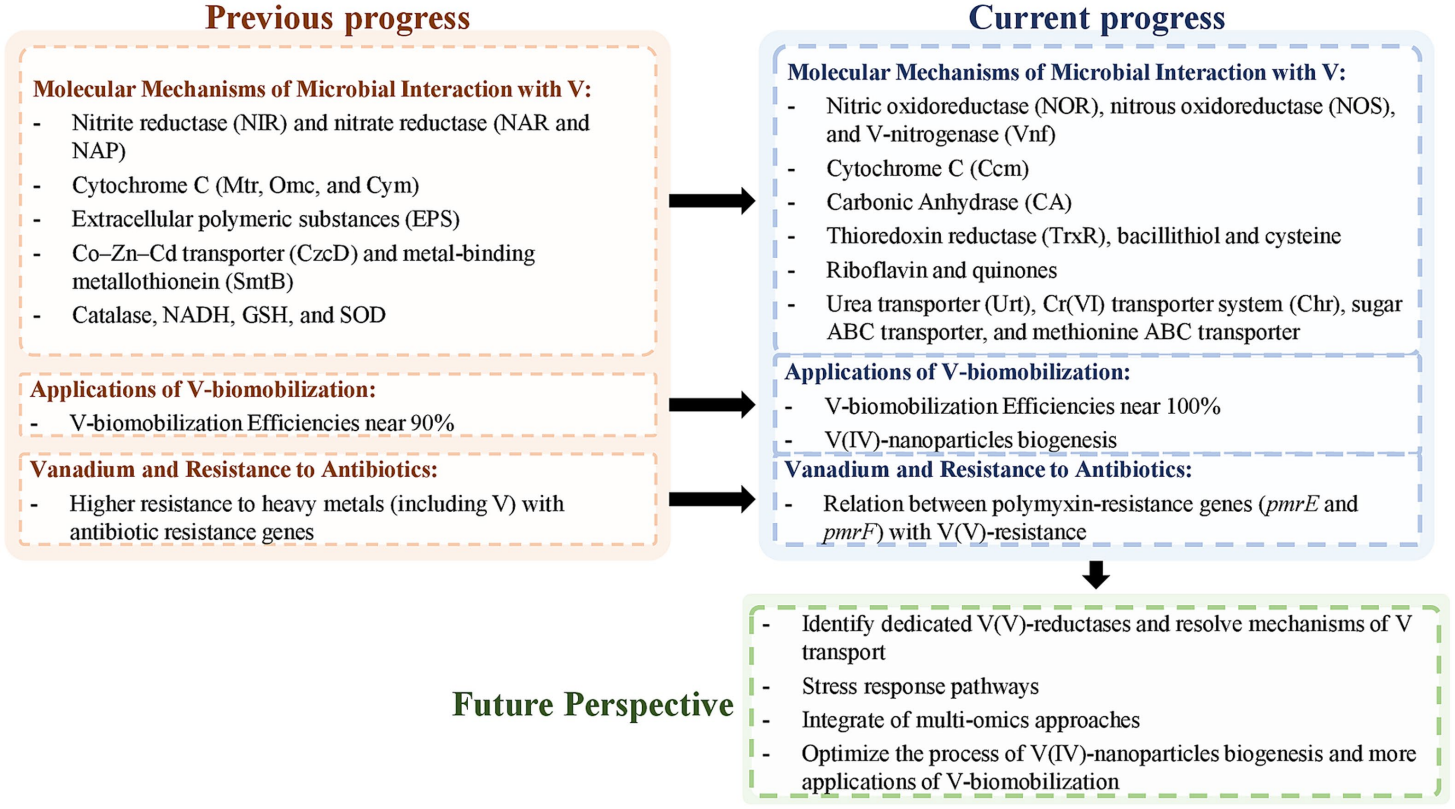
Schematic representation of past and current progress, along with future perspectives.

Significant progress has been made in understanding bacterial interactions with V, although several identified critical knowledge gaps remain, which limit both mechanistic insight and environmental application (Figure 2). One of the gaps identified is the lack of identified dedicated V(V)-reductases. The reviewed literature has shown that multiple bacteria can promote V(V)-bioreduction; however, no specific V(V)-reductase has been identified or biochemically characterized. It remains unclear whether V(V)-bioreduction is catalyzed by dedicated enzymes or occurs via activity of other oxidoreductases (nitrate, iron, or sulfur reductases). The identification, purification, and genetic validation of candidate enzymes represent a major priority for this field. Mechanisms of V transport and intracellular handling are also not clear. The molecular basis of V transport (uptake, efflux) remains poorly understood. Given the structural similarity between V(V) and other molecules, such as phosphate and nitrate, it is hypothesized that V may enter cells through the transport systems that normally handle these molecules. However, experimental conclusive confirmation is required to validate this hypothesis. Additionally, the specificity, regulation, and energetic costs associated with V transport systems require analysis. Future research could also focus on how these mechanisms are related to nanoparticles biogenesis. The mechanisms behind this process are still not known, so further exploration is needed to develop a green biotechnology for synthesizing V(IV)-nanoparticles.

The regulatory networks controlling V-tolerance and transformation are largely unknown and also constitute a gap. It is still unclear which stress regulators, redox-responsive transcription factors, or metal-sensing systems are involved in the response to V-exposure. More research is needed to clarify how V-exposure shapes cellular metabolism and stress adaptation. The integrative analysis of genomics, transcriptomics, proteomics, and metabolomics could reveal functional genes, identify metabolic bottlenecks, and clarify regulatory networks involved in V-cycling, toward systems-level understanding.

When considering V fate in natural environments, V transformation results from interactions within complex communities and probably not from single cell interaction. The roles of competition, synergy, and horizontal gene transfer in shaping V interactions and transformation capacity remain insufficiently explored.

## 9 Conclusion

**Figure 3:**
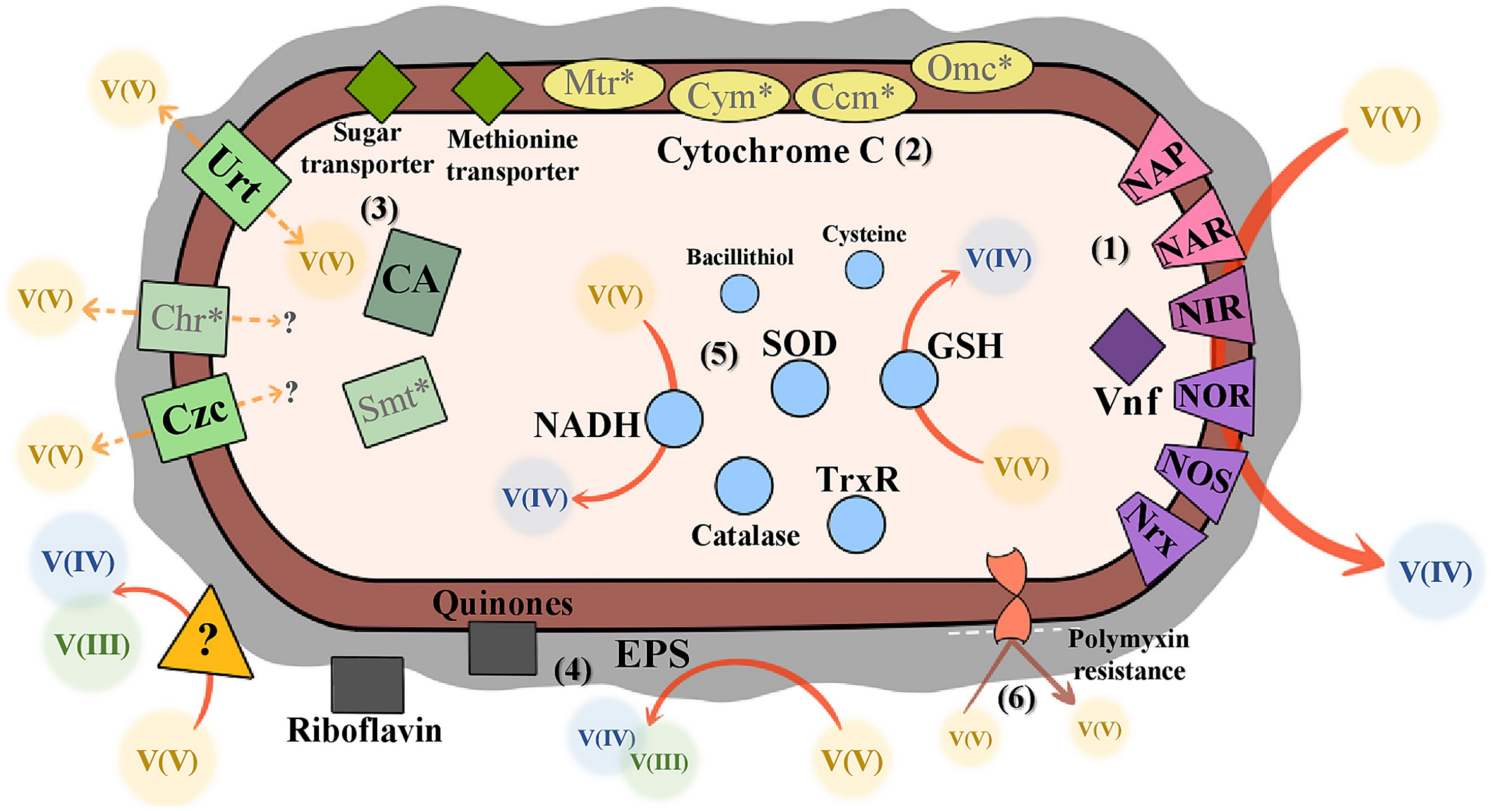
Illustrative representation of the possible molecular mechanisms for bacteria to cope with V-exposure: (1) denitrifying mechanisms—nitrate reductase enzymes NAP (*napA*, napAB*, and napB*) and NAR (*narG*), nitrite reductase enzyme NIR (nrfA, nirB, nirD, nirK, nirKS*, and *nirS*), nitric oxidoreductase NOR enzyme (norBC*), nitrous oxidoreductase enzyme NOS (*nosZ* and nrxAB*), and V-nitrogenase Vnf (*vnfA* and *vnfZ*); (2) cytochrome *c*-type—membrane cytochrome Mtr* (mtrA* and mtrC*), cytoplasmic membrane component Cym* (cymA*), cytochrome *c* maturation protein C* (ccmC*), and outer membrane cytochrome Omc* (omcA*, omcAB*, and omcB*); (3) resistance mechanisms to other metals and compounds—chromate transport protein Chr* (chrA*, chrAC*, and chrC*), cobalt–zinc–cadmium transporter Czc (*czcD*), metal-binding metallothionein Smt* (smtB*), urea ABC transporter Urt (*urtA, urtC,* and *urtE*), carbonic anhydrase CA, sugar (maltose/G3P/polyamine/iron) ABC transporter, and methionine ABC transporter; (4) extracellular secretions—extracellular polymeric substances (EPS), quinones and riboflavin (*apbE, FOXRED1, ribF, ribBA,* and *ribD*), and an undescribed protein; (5) stress mechanisms—reduced glutathione (GSH) (Gram-negative bacteria), NADH-dependent reductase, catalase, superoxide dismutase (SOD), bacillithiol (*bshBA, bshC, ypdA,* and *ytxJ*) (Gram-positive bacteria), cysteine (*cysJ*), and thioredoxin reductase TrxR (*trxA, trxR,* and *btuE*); (6) polymyxin resistance mechanisms (*pmrE* and *pmrF*). The molecular mechanisms marked with an asterisk (*) have been documented in the literature as existing within microbial communities; however, there is no description available in the analyzed literature for isolated strains or their activity quantification.

In conclusion, recent studies have sought to clarify the bacterial mechanisms employed to deal with V. These studies have provided a foundation for understanding the mechanisms that can be activated by exposure to this heavy metal (Figure 3). Although no specific gene or protein has been directly associated with V-resistance and V(V)-bioreduction, a strong relation has been established between V and several mechanisms. These include: (1) denitrification, (2) cytochrome c-type involving processes, (3) resistance to other heavy metals and compounds and the respective transporters, (4) extracellular secretions, (5) cellular stress responses, and (6) mechanisms for polymyxin resistance.

A collective analysis of existing literature and the mechanisms described suggests the potential for V(V)-bioreduction to occur both within and outside bacterial cells. This can be interpretated has a cell mechanism to reduce V-bioavailability, and therefore toxicity, since V(IV) is less soluble. The efflux of V is facilitated by ATP-binding cassette (ABC) transporters, which are known to transport other metals and compounds, such as Chr, responsible for Cr(VI) efflux; Czc, a protein described for Cd(II), Co(II) and Zn(II) extrusion; and Urt, a urea transporter. Moreover, V-uptake into bacterial cells may be linked to existing ABC transporters that manage other compounds and metals, such as the urea transporter Urt. Within the cells, a variety of processes can take place, thereby activating mechanisms that aid bacteria in their ability to cope with V at the intracellular level. In the cytoplasm, the V(V)-bioreduction process is predominantly catalyzed by NADH-dependent reductases and reduced glutathione. Once inside the cell, V triggers oxidative redox mechanisms typically associated with oxidative stress, involving enzymes such as superoxide dismutase (SOD), thioredoxin reductase (TrxR), and catalase. The specific redox mechanism can differ between bacterial type; for example, Gram-positive bacteria utilize bacillithiol (BSH), while Gram-negative bacteria use reduced glutathione (GSH). Furthermore, enzymes implicated in denitrification (namely, NAP, NAR, NIR, NOR, and NOS) facilitate the bioreduction of V(V). The process of nitrogen fixation may also be related to V, as certain nitrogenases require V as a catalyst, specifically V-nitrogenases (Vnf). Extracellularly, two main mechanisms are crucial for V(V)-bioreduction. The EPS has been shown to immobilize V onto the cell, thereby facilitating its bioreduction. In addition, unidentified proteins, which remain unstudied and uncharacterized, may also facilitate V(V)-bioreduction and potentially contribute to the formation of V(IV)-nanoparticles.
