# Supplementary material for: Understanding the role of vanadium: insights into bacterial responses and adaptations
Source: Front Microbiol. 2026 Apr 9;17:1788713. doi: 10.3389/fmicb.2026.1788713 (PMC13102585; doi:10.3389/fmicb.2026.1788713)
Supplement: Supplementary file 1 [file Supplementary_file_1.pdf]

Supplementary Material

Understanding the Role of Vanadium: Insights into Bacterial Responses and Adaptations

Joana B. Caldeira[1], Rita Branco[1], Paula V. Morais[1*]

[1] University of Coimbra, Centre for Mechanical Engineering, Materials and Processes, ARISE, Department of Life Sciences, 3000-456 Coimbra, Portugal

Supplementary Table 1. List of the 45 articles selected for the review.

Reference	Authors	Title	Year	Journal	Section
**(Almeida et al., 2020)**	Almeida, M. C., Branco, R., and Morais, P. V.	Response to vanadate exposure in *Ochrobactrum tritici* strains	2020	PLoS One	4+5
**(Appia-Ayme et al., 2022)**	Appia-Ayme, C., Little, R., Chandra, G., Martins, C. D., Batista, M. B., and Dixon, R.	Interactions between paralogous bacterial enhancer-binding proteins enable metal-dependent regulation of alternative nitrogenases in *Azotobacter vinelandii*	2022	Molecular Microbiology	4
**(Cheng et al., 2025)**	Cheng, M. M., Yin, X., and Zhang, H. K.	Insights into the hydrogen-fueled bioreduction of vanadium(V) by marine *Shewanella* sp. FDA-1: Process and mechanism	2025	Journal of Hazardous Materials	4+5
**(Chi et al., 2024)**	Chi, Z. F., Zhang, L. T., Ju, S. J., Li, W. J., Li, H., and Ren, X. Y.	Vanadium bioreduction in an ethane-based membrane biofilm reactor: Performance and mechanism	2024	Chemical Engineering Journal	5
**(Dong et al., 2024)**	Dong, Y. B., Zan, J. Y., and Lin, H.	Bioleaching of vanadium from stone coal vanadium ore by *Bacillus mucilaginosus*: Influencing factors and mechanism	2024	International Journal of Minerals, Metallurgy and Materials	4+5
**(Edet et al., 2023)**	Edet, U. O., Bassey, I. U., and Joseph, A. P.	Heavy metal co-resistance with antibiotics amongst bacteria isolates from an open dumpsite soil	2023	Heliyon	6
**(Fan et al., 2025)**	Fan, X. X., Zhang, Z., Zhou, W., Zhan, Y., Gao, S. C., and Liu, Y.	Enhanced performance of electricity generation and vanadium (V) reduction in microbial fuel cells via an efficient optimizing method	2025	Chemical Engineering Journal	5

**(Fei et al., 2023)**	Fei, Y. M., Zhang, B. G., Chen, D. D., Liu, T. X., and Dong, H. L.	The overlooked role of denitrifying bacteria in mediating vanadate reduction	2023	Geochimica et Cosmochimica Acta	4+5
**(Fei et al., 2024)**	Fei, Y. M., Zhang, B. G., Zhang, Q. H., Chen, D. D., Cao, W. E., and Borthwick, A. G. L.	Multiple pathways of vanadate reduction and denitrification mediated by denitrifying bacterium *Acidovorax* sp. strain BoFeN1	2024	Water Research	4+5
**(Fierros-Romero et al., 2020)**	Fierros Romero, G., Gómez-Ramírez, M., Sharma, A., Pless, R. C., and Rojas-Avelizapa, N. G.	*czcD* gene from *Bacillus megaterium* and *Microbacterium liquefaciens* as a potential nickel–vanadium soil pollution biomarker	2020	Journal of Basic Microbiology	4
**(Gan et al., 2024)**	Gan, C. D., Yang, J. Y., Li, J. F., Yang, M. Q., Du, X. Y., and Nikitin, A.	Transcriptome analysis reveals vanadium reduction mechanisms in a bacterium of *Pseudomonas balearica*	2024	Journal of Cleaner Production	4
**(Hao et al., 2021a)**	Hao, L. T., Liu, Y. J., Chen, N., Hao, X. D., Zhang, B. G., and Feng, C. P.	Microbial removal of vanadium (V) from groundwater by sawdust used as a sole carbon source	2021	Science of the Total Environment	5
**(Hao et al., 2021b)**	Hao, L. T., Zhang, B. G., Feng, C. P., Zhang, Z. Y., Lei, Z. F., and Shimizu, K. Y.	Human health risk of vanadium in farmland soils near various vanadium ore mining areas and bioremediation assessment	2021	Chemosphere	5
**(He et al., 2021)**	He, C., Zhang, B., Lu, J., and Qiu, R.	A newly discovered function of nitrate reductase in chemoautotrophic vanadate transformation by natural mackinawite in aquifer	2021	Water Research	4+5
**(He et al., 2023)**	He, J. X., Zhang, B. G., Wang, Y. N., Chen, S. M., and Dong, H. L.	Vanadate bio-detoxification driven by pyrrhotite with secondary mineral formation	2023	Environmental Science & Technology	4+5
**(Huang et al., 2025)**	Huang, Z. L., Wei, X. P., Zhang, L. J., Chen, T., Huang, Y., Yang, Z. W., Zeng, W. Q., Chen, H. X., Xie, L. T., and Yan, B.	Reduction of pentavalent vanadium via enhanced levels of sulfhydryl by elemental sulfur reducing bacteria from a stone coal mining region	2025	ACS ES&T Engineering	4+5
**(Jia et al., 2025)**	Jia, B. Y., Zhang, S. Y., Zhang, L. J., Li, W. J., Wang, Q. Y., Wan, J. J., Zeng, W. Q., Rossetti, S., Yan, B., and Su, X. T.	Unveiling the role of intermittent electrostimulation: enhancing microbial metabolism and electron transfer in electroactive biofilms to optimize V(V) reduction and immobilization	2025	ACS ES&T Engineering	5
**(Joo et al., 2023)**	Joo, H., Eom, H., Cho, Y., Rho, M., and Song, W. J.	Discovery and characterization of polymyxin-resistance genes *pmrE* and *pmrF* from sediment and seawater microbiome	2023	Microbiology Spectrum	6
**(Kim et al., 2025)**	Kim, B., Woo, D. K., Jeong, J., and Sim, M. S.	Vanadate reduction by gram-positive fermentative bacteria isolated from deep-sea sediments on the northern Central Indian Ridge	2025	PLoS One	4+5
**(Ko et al., 2025)**	Ko, Y., Kang, S., Yang, Y., Lee, J., and Hur, H. G.	Green synthesis of vanadium dioxide nanoparticles by *Shewanella* sp. strain HN-41	2025	Journal of Microbiology and Biotechnology	5

**(Komijani et al., 2021)**	Komijani, M., Shamabadi, N. S., Shahin, K., Eghbalpour, F., Tahsili, M. R., and Bahram, M.	Heavy metal pollution promotes antibiotic resistance potential in the aquatic environment	2021	Environmental Pollution	6
**(Li et al., 2022)**	Li, L., Zhang, B. G., Shi, J. X., He, J. X., Zhang, W., Yan, W. Y., Li, M., Tang, C. J., and Li, H. L.	Concurrent vanadate and ammonium abatement in a membrane biofilm reactor	2022	Chemical Engineering Journal	5
**(Liu et al., 2022)**	Liu, H., Chen, S. M., Lu, J. P., Li, Q. M., Li, J. L., and Zhang, B. G.	Pentavalent vanadium and hexavalent uranium removal from groundwater by woodchip-sulfur based mixotrophic biotechnology	2022	Chemical Engineering Journal	5
**(Liu et al., 2023)**	Liu, X., Pang, L., Yue, Y., Li, H., Chatzisymeon, E., Lu, Y., and Yang, P.	Insights into the shift of microbial community related to nitrogen cycle, especially N_2O in vanadium-polluted soil	2023	Environmental Pollution	4
**(Ma et al., 2024)**	Ma, R. Y., Feng, Y. L., Li, H. R., Liu, M. Y., Cui, Y. F., Wang, J. W., Shen, K. X., Zhang, S. B., and Tong, S. Z.	Deep-sea microorganisms-driven V^{5+} and Cd^{2+} removal from vanadium smelting wastewater: Bacterial screening, performance and mechanism	2024	Environmental Pollution	5
**(Mikoda et al., 2021)**	Mikoda, B., Potysz, A., Gruszecka-Kosowska, A., Kmiecik, E., and Tomczyk A.	Spent sulfuric acid plant catalyst: valuable resource of vanadium or risky residue? Process comparison for environmental implications	2021	Environmental Science and Pollution Research	5
**(Shi et al., 2020)**	Shi, C., Cui, Y., Lu, J., and Zhang, B.	Sulfur-based autotrophic biosystem for efficient vanadium (V) and chromium (VI) reductions in groundwater	2020	Chemical Engineering Journal	4+5
**(Stasiuk and Matlakowska, 2021)**	Stasiuk, R., and Matlakowska, R.	Postdiagenetic bacterial transformation of nickel and vanadyl sedimentary porphyrins of organic-rich shale rock (fore-sudetic monocline, Poland)	2021	Frontiers in Microbiology	5
**(Sun et al., 2020)**	Sun, X. X., Qiu, L., Kolton, M., Häggblom, M., Xu, R., Kong, T. L., Gao, P., Li, B. Q., Jiang, C. J., Sun, W. M.	V^{V} reduction by *Polaromonas* spp. in vanadium mine tailings	2020	Environmental Science & Technology	4+5
**(Sun et al., 2025)**	Sun, Z., Li, B., and Liu, J.	Synchronous vanadium bio-reduction/detoxification/recovery and nitrogen attenuation in a membrane aerated biofilm reactor	2025	Environmental Pollution	4+5
**(Tian et al., 2023)**	Tian, H., Cai, Z., Zhang, Y., and Zheng, Q.	Chemical mutation of *Bacillus mucilaginosus* genes to enhance the bioleaching of vanadium-bearing shale	2023	Biochemical Engineering Journal	5
**(Wang et al., 2023a)**	Wang, H. S., Chen, N., and Feng, C. P.	Priming effect and mechanism of nitrate and vanadate removal from agro-industrial waste-based colonizing microbial communities	2023	Journal of Cleaner Production	5
**(Wang et al., 2023b)**	Wang, S. X., Zhang, B. G., Fei, Y. M., Liu, H., Zhao, Y., and Guo, H. M.	Elucidating multiple electron-transfer pathways for metavanadate bioreduction by Actinomycetic *Streptomyces microflavus*	2023	Environmental Science & Technology	5

**(Wang et al., 2021)**	Wang, Z. L., Zhang, B. G., He, C., Shi, J. X., Wu, M. X., and Guo, J. H.	Sulfur-based mixotrophic vanadium (V) bio-reduction towards lower organic requirement and sulfate accumulation	2021	Water Research	4+5
**(Yan et al., 2023)**	Yan, W. Y., Chen, S. M., Li, M., Liu, S. T., and Zhang, B. G.	Genome-resolved metagenomic insight into vanadate and ammonium elimination in sulfur-based autotrophic biosystem	2023	Chemical Engineering Journal	4+5
**(Yan et al., 2025)**	Yan, W. Y., Zhang, B. G., Li, Y. N., Lu, J. P., Dong, H. L., Fei, Y. M., Zhou, S. G., and Huang, F.	Electron transfer pathways and vanadium isotope fractionation during microbially mediated vanadate reduction	2025	Engineering	4+5
**(Yuliani et al., 2024)**	Yuliani, D., Morishita, F., Imamura, T., and Ueki, T.	Vanadium accumulation and reduction by vanadium-accumulating bacteria isolated from the intestinal contents of *Ciona robusta*	2024	Marine Biotechnology	5
**(Zhang et al., 2025a)**	Zhang, B. G., Fei, Y. M., Diao, M. H., Liu, T. X., Shaheen, S. M., Rinklebe, J., Zhou, S. G., Dong, H. L., and Ren, Z. J.	Trivalent vanadium precipitation in siderite-dependent vanadate bioreduction by denitrifying bacteria in groundwater	2025	Environmental Science & Technology	4
**(Zhang et al., 2020)**	Zhang, B. G., Jiang, Y. F., Zuo, K. C., He, C., Dai, Y. R., and Ren, Z. J.	Microbial vanadate and nitrate reductions coupled with anaerobic methane oxidation in groundwater	2020	Journal of Hazardous Materials	5
**(Zhang et al., 2021a)**	Zhang, B. G., Li, Y. N., Fei, Y. M., and Cheng, Y. T.	Novel pathway for vanadium(V) bio-detoxification by gram-positive *Lactococcus raffinolactis*	2021	Environmental Science & Technology	4+5
**(Zhang et al., 2022)**	Zhang, H., Shi, J. X., Chen, C. B., Yang, M., Lu, J. P., and Zhang, B. G.	Heterotrophic bioleaching of vanadium from low-grade stone coal by aerobic microbial consortium	2022	International Journal of Environmental Research and Public Health	5
**(Zhang et al., 2021b)**	Zhang, H., Zhang, B. G., Gao, Y. Q., Wang, Y., Lu, J. P., Chen, J. L., Chen, D. D., and Deng, Q. L.	The role of available phosphorous in vanadate decontamination by soil indigenous microbial consortia	2021	Environmental Pollution	5
**(Zhang et al., 2025b)**	Zhang, L. J., Yang, Z. W., Zeng, W. Q., Liang, B. Y., Huang, Z., Wang, Q. Y., Li, Z. M., Chen, T., and Yan, B.	Hierarchical vanadium detoxification mechanisms in gram-positive *Enterococcus faecalis*: extracellular chelation, antioxidant defense, and metabolic reprogramming	2025	ACS ES&T Engineering	4+5
**(Zheng et al., 2021)**	Zheng, X., Zhao, B., and Liu, C. G.	Bio-reduction mechanism of V(V) by thermophilic hydrogen-producing bacteria under acidic conditions	2021	Environmental Science: Water Research & Technology	4+5
**(Zhou et al., 2022)**	Zhou, D., Liang, M. M., Xia, Y. L., Li, C., Huang, M. Z., Peng, S. M., and Huang, Y.	Reduction mechanisms of V^{5+} by vanadium-reducing bacteria in aqueous environments: role of different molecular weight fractionated extracellular polymeric substances	2022	Science of the Total Environment	4+5
